# Na^+^ extrusion from the cytosol and tissue-specific Na^+^ sequestration in roots confer differential salt stress tolerance between durum and bread wheat

**DOI:** 10.1093/jxb/ery194

**Published:** 2018-06-11

**Authors:** Honghong Wu, Lana Shabala, Elisa Azzarello, Yuqing Huang, Camilla Pandolfi, Nana Su, Qi Wu, Shengguan Cai, Nadia Bazihizina, Lu Wang, Meixue Zhou, Stefano Mancuso, Zhonghua Chen, Sergey Shabala

**Affiliations:** 1 School of Land and Food, University of Tasmania, Private Bag, Hobart, Tasmania, Australia; 2 Department of Horticulture, University of Florence, Sesto Fiorentino, Italy; 3 School of Science and Health, Hawkesbury Institute for the Environment, Western Sydney University, Penrith, NSW, Australia; 4 School of Natural Sciences, University of Tasmania, Private Bag 55, Hobart, Tasmania, Australia

**Keywords:** Long-distance signalling, sodium extrusion, sodium sensing, tissue specificity, vacuolar sequestration

## Abstract

The progress in plant breeding for salinity stress tolerance is handicapped by the lack of understanding of the specificity of salt stress signalling and adaptation at the cellular and tissue levels. In this study, we used electrophysiological, fluorescence imaging, and real-time quantitative PCR tools to elucidate the essentiality of the cytosolic Na^+^ extrusion in functionally different root zones (elongation, meristem, and mature) in a large number of bread and durum wheat accessions. We show that the difference in the root’s ability for vacuolar Na^+^ sequestration in the mature zone may explain differential salinity stress tolerance between salt-sensitive durum and salt-tolerant bread wheat species. Bread wheat genotypes also had on average 30% higher capacity for net Na^+^ efflux from the root elongation zone, providing the first direct evidence for the essentiality of the root salt exclusion trait at the cellular level. At the same time, cytosolic Na^+^ accumulation in the root meristem was significantly higher in bread wheat, leading to the suggestion that this tissue may harbour a putative salt sensor. This hypothesis was then tested by investigating patterns of Na^+^ distribution and the relative expression level of several key genes related to Na^+^ transport in leaves in plants with intact roots and in those in which the root meristems were removed. We show that tampering with this sensing mechanism has resulted in a salt-sensitive phenotype, largely due to compromising the plant’s ability to sequester Na^+^ in mesophyll cell vacuoles. The implications of these findings for plant breeding for salinity stress tolerance are discussed.

## Teaser

Stronger Na^+^ extrusion and vacuolar sequestration are essential to confer better salt tolerance in bread wheat than in durum wheat. Removal of the root meristems increased salt sensitivity in wheat.

## Introduction

Salinity stress is one of the major environmental constraints limiting agricultural crop production. However, physiological and genetic complexity of salinity stress tolerance has significantly handicapped the progress in crop breeding for salt-tolerant crops. One of the keys to overcoming this limitation is an understanding of the specificity of salt stress signalling and adaptation at the cellular and tissue levels.

Bread wheat (*Triticum aestivum*) is always considered as a more salt-tolerant species than durum wheat (*Triticum turgidum*) (Munns and James, 2003; Munns and Tester, 2008); this difference has been largely attributed to its superior ability to maintain lower Na^+^ accumulation in the leaf/shoot (Colmer *et al.*, 2006; Cuin *et al.*, 2010; James *et al.*, 2011; Munns *et al.*, 2012). However, it remains unclear whether this pattern is achieved by stronger Na^+^ exclusion traits from the shoot such as preventing Na^+^ loading into the xylem (Davenport *et al.*, 2005; Läuchli *et al.*, 2008; Zhu *et al.*, 2016) or increased Na^+^ retrieval from the shoot (James *et al.*, 2006; Davenport *et al.*, 2007), or is due to reduced net root Na^+^ uptake *per se*. Also, some recent studies showed the lack of significant correlation between shoot Na^+^ exclusion ability in a broad range of plants (Arabidopsis, Rus *et al.*, 2006; Jha *et al.*, 2010; wheat, Genc *et al.*, 2007; tomato, Almeida *et al.*, 2014), indicating the importance of Na^+^ sequestration in the shoot (so-called tissue tolerance) as arguably the most essential component of salinity stress tolerance (Munns *et al.*, 2016).

If the rate of unidirectional Na^+^ uptake in plants is about the same but they accumulate different quantities of Na^+^ in their tissues, then the difference in salinity stress tolerance may be attributed to the ability of a specific genotype to extrude Na^+^ actively after it has crossed the plasma membrane and entered the cytosol. This extrusion ability is mediated by the operation of the plasma membrane Na^+^/H^+^ exchangers encoded by *SOS1* (*Salt Overly Sensitive 1*) in Arabidopsis (Shi *et al.*, 2000, 2003). It is estimated that ~95% of all Na^+^ taken up by roots is pumped back into the rhizosphere (Munns and Tester, 2008) via this mechanism. To the best of our knowledge, the comparative functional analysis of the activity of SOS1-like Na^+^ efflux systems has never been conducted for a large number of plant accessions, and the question of whether the difference in salinity stress tolerance between durum and bread wheat is attributed to the difference in SOS1 activity has not been addressed. Meanwhile, insights into this issue might be beneficial for improving plant salt tolerance since in most cases the accumulated excessive Na^+^ in the cytosol causes the Na^+^ toxicity. Filling this knowledge gap was one of objectives of this study.

While the importance of cell type specificity in plant adaptive responses to salinity is widely accepted, most studies addressed this issue at the transcriptional (e.g. tissue-specific expression patterns of genes in plants under salt stress; Claes *et al.*, 1990; Löw *et al.*, 1996; Saijo *et al.*, 2000; Jain *et al.*, 2007; Dinneny *et al.*, 2008; Iyer-Pascuzzi *et al.* ,2011) rather than the functional level. At the same time, there is a growing body of evidence suggesting that tissue specificity of stress sensing may be a key to understanding the complexity of the overall plant salt tolerance (Kiegle *et al.*, 2000; Verdoy *et al.*, 2004; Wu *et al.*, 2015*a*; L. Shabala *et al.*, 2016). In this context, knowledge of Na^+^ distribution between various cell compartments (i.e. the cytosol and vacuole) in different cell types may shed light on both mechanisms enabling plant adaptation to saline conditions and the nature of the elusive Na^+^ sensor. Indeed, the importance of vacuolar Na^+^ sequestration for overall salt tolerance is widely accepted (Apse *et al.*, 1999; Zhang *et al.*, 2001), and improved salt tolerance resulting from overexpressing the NHX1 K^+^(Na^+^)/H^+^ exchanger has been reported in many species (Apse *et al.*, 1999; Xue *et al.*, 2004; H. Chen *et al.*, 2007; He *et al.*, 2007; Zhang and Blumwald, 2001). However, most of the reported studies were focused on the essentiality of vacuolar Na^+^ sequestration in the shoot mesophyll cells, while the patterns of Na^+^ uptake, distribution, and sequestration in various types of root cells remain largely unexplored. At the same time, root cells confer the first line of defence and have to deal with the presence of high Na^+^ concentrations in the rhizosphere. Is vacuolar Na^+^ sequestration in roots essential for salinity stress tolerance? If yes, can this conclusion be extrapolated to all cell types? Answering these questions was another objective of this study.

While downstream targets and effectors mediating plant adaptive responses to salinity have been studied in sufficient detail, the nature of the putative salt sensor in plants remains a great mystery (Maathuis, 2014; Shabala *et al.*, 2015). In our previous study (Wu *et al.*, 2015*a*), we compared several bread wheat genotypes contrasting in salinity tolerance and found that the salt-tolerant group possesses a higher fluorescence Na^+^ signal from the cytosol in the root meristem zone compared with the salt-sensitive group. This (unexpected) finding led to the suggestion that the root meristem zone might participate in salt sensing, or execute a role as a salt sensor. Validating this hypothesis was the third objective of this study.

The main findings of our work are 2-fold. First, we provide direct evidence that higher Na^+^ extrusion ability in the root elongation zone and better vacuolar Na^+^ sequestration ability in the mature root zone may be essential for superior salinity tolerance in bread wheat as compared with durum wheat. Secondly, we provide new supporting evidence that root meristem cells might act as a tentative salt sensor or at least harbour the salt sensor components, and tampering with this sensing mechanism in roots affects a plant’s ability to sequester Na^+^ in the shoot mesophyll via some as yet unknown long-distance signal propagating from the root to the shoot.

## Materials and methods

### Plant material and growth conditions for MIFE and laser scanning confocal microscopy (LSCM) experiments

We used 27 bread wheat (*Triticum aestivum*) and 19 durum wheat (*Triticum turgidum* spp. durum) varieties. All seeds were obtained from the Australian Winter Cereal collection and multiplied in our laboratory. Twenty seeds for each variety were surface sterilized with 5% commercial bleach (containing 42 g l^–1^ NaClO) for 10 min, and then washed under running tap water for ~0.5 h. From 12 to 16 uniform seeds were grown in a l litre PVC container with distilled water. Good aeration was provided with aquarium stones connected to an air pump (Aqua One^®^ 9500, Sydney, Australia). Seedlings were grown in darkness at 25 ± 2 °C for 3 d followed by the salinity treatment (100 mM NaCl) for 24 h. Salt-stressed seedlings were used to investigate the Na^+^ extrusion ability of wheat roots. The reasons why we used young wheat seedlings for microelectrode ion flux estimation (MIFE) experiments are 4-fold. First, root sensitivity to salt gradually declines with age (e.g. Chen *et al.*, 2005). Thus, working with young seedlings has the advantage of achieving a better signal-to-noise resolution (hence, more accurate genotypic differentiation). Secondly, the ultimate aim of this work was to arm breeders with convenient high-throughput tools allowing a rapid screening of hundreds of lines. Working with younger seedlings is more convenient from this perspective. Thirdly, the MIFE measurements require roots to be exposed to the aqueous medium. From our past experience, prolonged hydroponic plant growth in nutrient-rich solution is confounded by a high probability of roots being contaminated. Growing plants in the soil and then washing roots to make them accessible for MIFE measurements is also a lengthy and highly complicated process that is further jeopardized by the possibility of the root tissue being damaged by such procedures. Finally, preliminary experiments in our laboratory have shown that the observed genotypic difference in ion flux patterns is largely invariant with plant age (data not shown).

### Glasshouse experiments

Plants were grown in the glasshouse facilities at the University of Tasmania, essentially as described in Wu *et al.* (2015*b*). Briefly, 12–14 seeds of each variety were sown in a 4.5 litre PVC pot filled with standard potting mix (triplicates). After 6 d, pots were thinned to leave eight similar plants per pot, and salt treatment (300 mM NaCl) was applied for a further 5 weeks. A saucer was placed under each pot, and plants were irrigated twice a day by an automatic watering system with dripper outlets for both non-saline conditions and salinity treatment. The salt tolerance index (quantified on 0–10 scales and based on the extent of leaf chlorosis and the plant survival rate; Zhou *et al.*, 2012) was scored before harvesting (see Supplementary Fig. S1A at *JXB* online). The higher numbers represent the lower salinity tolerance (e.g. 0, no visual stress symptoms; 10, dead plants). The above tolerance index measured at the vegetative stage showed significant (*P*<0.001) correlation with the relative grain yield of wheat cultivars reported in our earlier experiments (Supplementary Fig. S1B) and thus could be used as a convenient proxy for quantification of plant salinity stress tolerance.

### Preparation of ion-selective microelectrodes for non-invasive ion flux measurement

Net Na^+^ and H^+^ fluxes were measured from excised roots using non-invasive MIFE (Shabala *et al.* 2006). Briefly, blank microelectrodes were pulled out from borosilicate glass capillaries (GC 150-10; Harvard Apparatus, Kent, UK), dried in an oven (at 225 ºC, overnight), and silanized with tributylchlorosilane (No 282707, Sigma-Aldrich, St. Louis, MO, USA). The silanized microelectrode blanks were filled from the back with an appropriate backfilling solution (for H^+^, 15 mM NaCl+40 mM KH_2_PO_4_, pH 6.0 adjusted using NaOH; and for Na^+^, 0.5 M NaCl) followed by front filling the tip with a corresponding liquid ion exchanger (LIX). A commercially available LIX was used for H^+^ (catalogue no. 95297, Sigma-Aldrich) while the LIX for Na^+^ was prepared in the laboratory as described in Jayakannan *et al.* (2011). The prepared microelectrodes were mounted in MIFE electrode holders and calibrated in an appropriate set of standards (see Shabala *et al.*, 2006 for methodological details). Only microelectrodes with a slope >50 mV per decade and correlation >0.999 were used.

### MIFE screening for root Na^+^ extrusion ability

All 46 bread and durum wheat genotypes were screened for their ability to extrude Na^+^ actively from the root, using the non-invasive MIFE technique. For this, we have used the protocol developed by Cuin *et al.* (2011) and validated in further studies (Zhu *et al.*, 2016). In brief, roots of hydroponically grown seedlings treated with 100 mM NaCl for 24 h [see ‘Plant material and growth conditions for MIFE and laser scanning confocal microscopy (LSCM) experiments’] were gently rinsed in a beaker containing 10 mM CaCl_2_ for 1 min followed by gentle rinsing in ddH_2_O for another minute. About 3 cm long root segments were cut starting from the root tip. Excised root segments were then mounted in a fixed plastic holder in a Petri dish containing 10 ml of ddH_2_O. Na^+^ and H^+^ selective microelectrode tips were positioned 40 μm above the root epidermis in the middle of the elongation zone (~2 mm from the tip). Measurements started 30 min after immobilization. By this time, all transient responses related to Donnan exchange in the apoplast have subsided (see Cuin *et al.*, 2011 for evidence) and the measured Na^+^ efflux from the root was mediated mainly by SOS1-like Na^+^/H^+^ exchangers in the root plasma membrane (see Supplementary Fig. S2 for supporting evidence). During measurements, electrodes were travelling forth and back between two positions, 40 μm and 110 μm from the root surface, in a 12 s square-wave cycle. The electrochemical gradient potential was recorded and then converted into net ion fluxes using the calibration values of the ion-selective microelectrodes and cylindrical diffusion geometry of the root (MIFEFLUX software; Newman, 2001; Shabala *et al.*, 2006). At least six root segments were assessed for each treatment and variety. Each root was measured for at least 5 min, to ensure steady-state flux values.

### Imaging Na^+^ distribution in vacuolar and cytosolic compartments in mesophyll cells and different root zones in wheat by LSCM

The green fluorescent Na^+^ dye CoroNa Green acetoxymethyl ester (cat. no. C36676, Invitrogen) was used to assess the amount of Na^+^ accumulated in the cytosol and vacuole in different root zones and also mesophyll cells using established protocols (Bonales-Alatorre *et al.*, 2013 for mesophyll cells; Wu *et al.*, 2015*a* for root cells). For root measurements, two 10 mm long segments were cut from seminal wheat roots—one in the mature zone (30–40 mm from the apex) and another one from the apex (the first 10 mm). Four-day-old wheat seedlings (grown hydroponically) under 100 mM NaCl for another 72 h were used for collecting root samples. For the leaf experiment, the youngest fully expanded leaves were excised, their abaxial epidermis peeled off with a pair of fine forceps to expose the mesophyll cells, and 5 × 8 mm segments were cut. Leaf samples were prepared from plants grown in the potting mix. Roots meristem tissue was removed from 4-day-old hydroponically grown seedlings. These seedlings were then transplanted to the potting mix, allowed to adapt for 2 d, and then treated with 200 mM NaCl for 2 weeks. Root and leaf samples were incubated in a solution containing 20 μM CoroNa Green for 2 h in the dark, rinsed in a buffer solution (5 mM MES, pH 6.3 adjusted with KOH), and examined using an upright Leica laser scanning confocal microscope SP5 (Leica Microsystems, Germany) equipped with a ×40 oil immersion objective (excitation wavelength, 488 nm; emission, 510–520 nm). LAS Lite software (Leica Microsystems, Germany) was used for image analyses (Supplementary Fig. S3).

To validate the above protocol further and provide accurate quantification of the fluorescent signal in each intracellular compartment, roots were co-stained with CoroNa Green-AM and FM4-64, a dye that stains membrane structures including the plasma and tonoplast mebranes (Oh *et al.*, 2010; Bassil *et al.*, 2011). Roots were co-stained with 20 μM CoroNa Green-AM and 20 μM FM4-64 for 2 h in the dark as describe above. The samples were then rinsed with the buffer solution (5 mM MES, pH 6.3 adjusted with KOH) for 3 min and analysed using confocal imaging facilities (Confocal SP5). The 488 nm excitation line was used for FM4-64 fluorescence, and its signals were collected with a 615 nm long-pass filter. Figure 4 shows the results of the co-staining of roots with CoroNa Green dye and FM4-64 membrane dye.

As CoroNa Green is not a ratiometric dye, the absolute Na^+^ concentration in cell compartments could not be quantified. Because of this, the mean fluorescence intensity values for cytosolic and vacuolar compartments were calculated (in arbitrary units) for each cell in each region. Light microscopy images (visualizing root cell morphology) and FM4-64 membrane dye were used to clarify the signal profile distribution in the cytosol and vacuole, and special attention was paid to ensure that all the imaging settings on the confocal microscope were identical, to allow such a comparison. Root meristem, elongation, and mature zones were distinguished by root morphology. Readings (72–96 cells for each genotype for the root experiment and 47–86 cells for the mesophyll experiment) were averaged and reported. This large sample size has minimized possible confounding effects of dye loading. It should also be mentioned that we have previously undertaken a comprehensive methodological study optimizing the loading time and dye concentration (Wu *et al.*, 2015*a*) and showed that reported readings are not affected by any salt stress-induced fluctuation in either cytosolic pH or K^+^ (Wu *et al.*, 2015*a*).

Before the above experiments were conducted, the possible impact of the loading time and dye concentration used was studied in dedicated methodological experiments. In one of them, we studied the effect of CoroNa Green dye concentration on observed patterns of Na^+^ distribution within the cell (using a dye concentration up to 80 μM). These results are shown in Supplementary Fig. S4. We have also studied the effects of incubation time (increasing the time of dye loading up to 5 h; Supplementary Fig. S5). Our results (Supplementary Figs S4, S5) showed that the patterns of Na^+^ signal distribution between the cytosol and vacuole in the root zones of two contrasting varieties were invariant in both dye concentration used and loading (incubation) time. Also, the pattern of Na^+^ intensity between the cytosol and vacuole in wheat root is not affected by a different depth of laser scanning (Wu *et al.*, 2015*a*).

### Studying physiological effects of removal of the root meristem

From 12 to 16 uniform seeds of salt-tolerant bread wheat (variety Kharchia 65) were grown hydroponically in l litre PVC containers. The container was filled with ddH_2_O (sterile double-distilled water) and seedlings were grown with aeration for 3–4 d in the dark. Seedling numbers were thinned to six in each container. Roots meristem (the first ~0.3 mm apical segment from the root tip) was surgically removed from seminal roots in each plant under a microscope before applying treatment (Supplementary Fig. S6). Plants were then transferred back into containers with 1/4 Hoagland solution and grown for a further 10 d in the presence and absence of 200 mM NaCl under ~150 μmol m^–2^ s^–1^ irradiance at 26/20 ± 1.0 °C day/night temperatures, 16 h/8 h day/night cycle. A number of physiological parameters were measured at the end of the experiment, including: leaf chlorophyll content (measured in arbitrary units by a chlorophyll meter SPAD-502, Konica Minolta, Tokyo, Japan), the maximal photochemical efficiency of PSII, *F*_v_/*F*_m_ (quantified by a chlorophyll fluorometer OS-30p, Optisciences, Hudson, NH, USA), and shoot and root FW (from plants rinsed in ddH_2_O to remove the possible salt residues). Samples were then dried at 65 °C for 72 h in a Unitherm Dryer (Birmingham, UK) and the DW of shoots and roots was estimated. During salt stress treatment, lateral roots in salt-stressed plants were checked and eliminated every day.

Another experiment with root meristem removal was conducted in plants grown in a potting mix under glasshouse conditions. Seedlings were grown in paper rolls in containers with ddH_2_O for 4 d. Root meristem segments were removed from each root of each plant, as described above. Then control plants (with intact roots) and cut plants (with the meristem zone removed from roots) were transplanted to a 1.7 litre pot filled with the standard potting mix [80% composted pine bark; 10% sand and 10% coir peat; plus complete N:P:K (8:4:10), 1 kg m^–3^; dolomite, 8 kg m^–3^; gypsum, 1 kg m^–3^; iron sulphate, 1 kg m^–3^; isobutylenediurea, 1 kg m^–3^; trace element mix, 0.75 kg m^–3^; wetting agent, 0.75 kg m^–^; zeolite, 0.75 kg m^–3^; pH 6.0]. Pots were hand-watered with tap water for 2 d to allow the transplanted plants to adapt to the potting mix. Then, plant numbers were thinned to six in each pot and salt stress (200 mM NaCl) was applied for a further 15 d. At the end of the experiment, SPAD values were measured.

### Leaf and root Na^+^ content

Dry leaf and root samples were digested in a 120 ml Teflon digestion vessel in a microwave digester (Mars 6 Microwave Digestion System, CEM Corporation, Matthews, NC, USA) using 7 ml of 70% HNO_3_ per 0.1 g specimen. Digested samples were centrifuged at 5000 *g* for 10 min at room temperature (Avanti J-30I centrifuge, Beckman Coulter, Krefeld, Germany). After centrifugation, 0.2 ml of the digested solution was diluted with ddH_2_O to a final volume of 10 ml. Na^+^ content was then measured using a Flame Photometer (PFP7, Jenway, UK).

### Quantitative real-time PCR analysis

Plants with intact and cut roots grown in 1/4 Hoagland solution in the absence and presence of 200 mM NaCl were used. The youngest fully expanded leaves (variety Kharchia 65) were harvested and snap-frozen in liquid nitrogen. The leaf RNA was extracted and synthesized to cDNA by using the RNeasy Mini Kit (QIAGEN) and the QuantiTect^®^ Reverse Transciption Kit (QIAGEN) following the manufacturer’s instructions. Quantitative real-time PCR was performed using a RG6000 Rotor-Gene Q Real Time Thermal Cycler and SYBR green PCR reagent (QIAGEN) as described in Vandesompele *et al.* (2002). The 2^−∆∆C^_T_ method (Livak and Schmittgen 2001) was used to analyse the relative expression levels of the studied genes. Primers with a single melting curve were designed by Primer Premier6 based on the conserved part of the gene sequence to determine the expression of *TaSOS1*, *TaNHX1*, *TaVP*, and *TaHA1* (see Supplementary Table S1 for primer sequences). The control gene (*TaActin*) which shows a constant expression level in all samples was used for normalization of the test gene transcript. Experiments were repeated three times (each biological replicates had 3–4 technical replicates) with consistent results.

### Data analysis

All data (given as mean ±SE, *n*=biological replicates) were analysed by using SPSS 20.0 for windows (SPSS Inc., Chicago, IL, USA). The comparisons of different parameters between bread and durum wheat were performed by independent samples *t*-test and one-way ANOVA based on Duncan’s multiple range test (two tailed). *, **, and *** indicate *P*<0.05, *P*<0.01, and *P*<0.001, respectively; ns stands for *P*>0.05. Different lower case letters represent the significance at *P*<0.05. The significance of correlations between different parameters was determined by bivariate correlations based on Pearson correlation (two tailed).

## Results

### Bread wheat excludes more Na^+^ from the root elongation zone

**Fig. 1.**
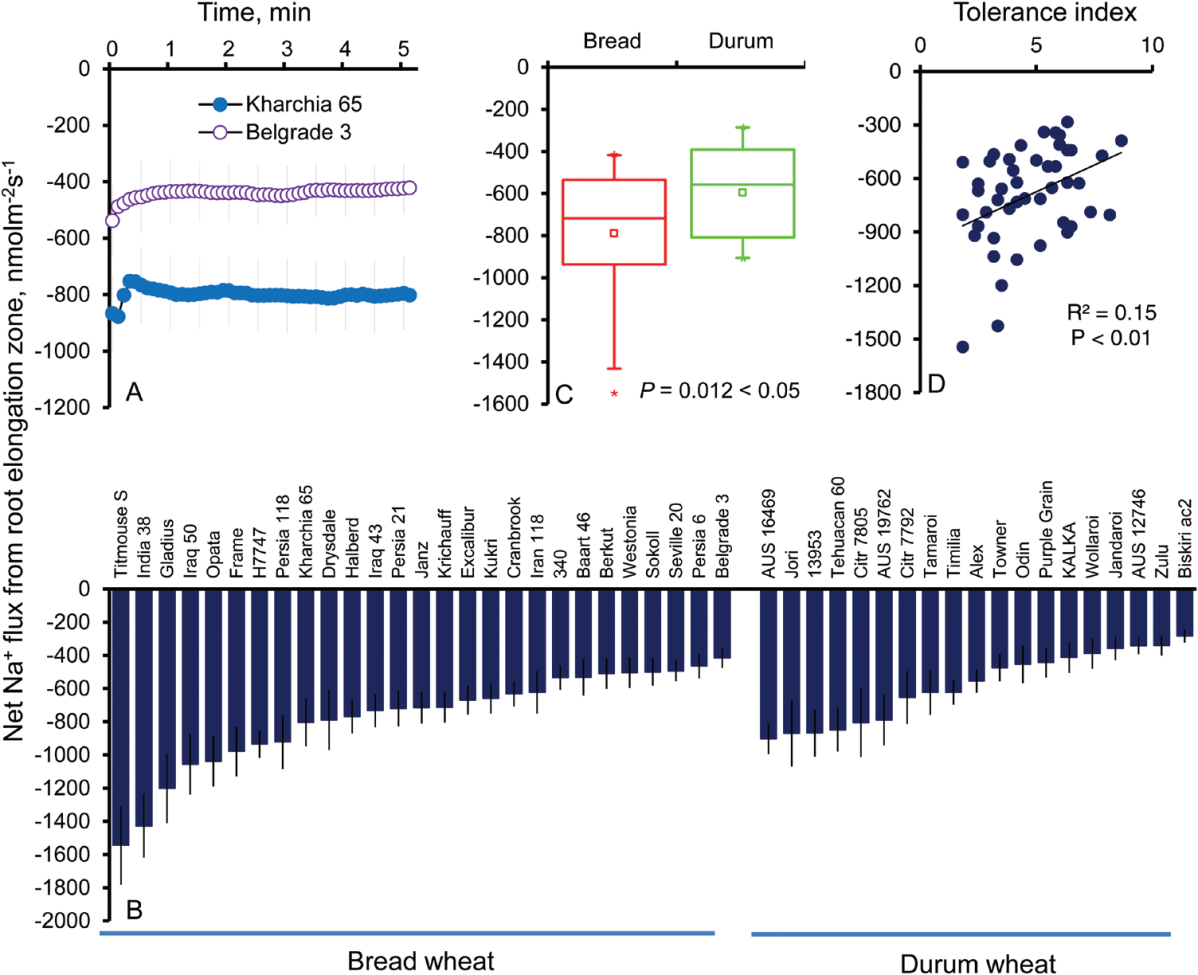
Genetic variability of Na^+^ efflux in the root elongation zone after the removal of external salt. (A) Net steady-state Na^+^ efflux measured from the root elongation zone after the removal of external salt in two bread wheat genotypes (Kharchia 65, salt tolerant; and Belgrade 3, salt sensitive). The sign convention is ‘efflux negative’. Mean ±SE (*n*=6−13). (B) Net steady-state Na^+^ efflux measured from all 46 wheat varieties. Mean ±SE (*n*=6−13). (C) Pooled mean values of net Na^+^ efflux for bread and durum wheat clusters. Mean ±SE [*n*=19 (durum wheat) and 27 (bread wheat)]. *Significant at *P*<0.05. (D) Correlation between net Na^+^ efflux and tolerance index in the studied wheat varieties. Every single point represents one variety. Hydroponically grown 3-day-old wheat seedlings treated with 100 mM NaCl for 24 h were used.

Confocal imaging of SOS1–green fluorescent fusion proteins in transgenic Arabidopsis plants indicated that SOS1 is localized in the plasma membrane, with the strongest signal detected in the root apex and, specifically, the elongation zone (Shi *et al.*, 2002). Accordingly, we have undertaken the functional assessment of SOS1-like activity in epidermal cells in this zone in various durum and bread wheat cultivars using the MIFE technique. At 30 min after rinsing with CaCl_2_ solution, measured Na^+^ fluxes were steady (Fig. 1A) and thus reflected the functional activity of SOS1. Two lines of evidence support this claim: (i) such Na^+^ efflux showed high sensitivity to amiloride, a known inhibitor of Na^+^/H^+^ exchangers (Kleyman and Cragoe, 1988; Cuin *et al.*, 2011); and (ii) unlike wild-type Arabidopsis plants, where Na^+^ efflux was detected, the process was absent from roots of the *sos1* mutant lacking a functional SOS1 transporter (Supplementary Fig. S2). Over 5-fold variability was found among the 46 screened wheat varieties, with the highest Na^+^ efflux being –1584 ± 237 (in Titmouse S, bread wheat) and the lowest Na^+^ efflux being –284 ± 39 nmol m^–2^ s^–1^ (in Biskiri AC2, durum wheat) (Fig. 1B). On average, Na^+^ extrusion ability in bread wheat in the root elongation zone was 33% higher compared with durum wheat (mean flux values 773 ± 55 nmol m^–2^ s^–1^ and –580 ± 48 nmol m^–2^ s^–1^; significant at *P*<0.05) (Fig. 1C). A strong and positive correlation was observed between Na^+^ extrusion and plant overall salinity stress tolerance based on the damage index (*r*=0.39, *P*<0.01; Fig. 1D). Also, a significant (*P*<0.01) up-regulation of *TaSOS1* in the root apex was found in bread wheat but not durum wheat under salt stress (Supplementary Fig. S7A).

### Durum wheat compensates poor Na^+^ extrusion ability from the root elongation zone by more efficient vacuolar Na^+^ sequestration

**Fig. 2.**
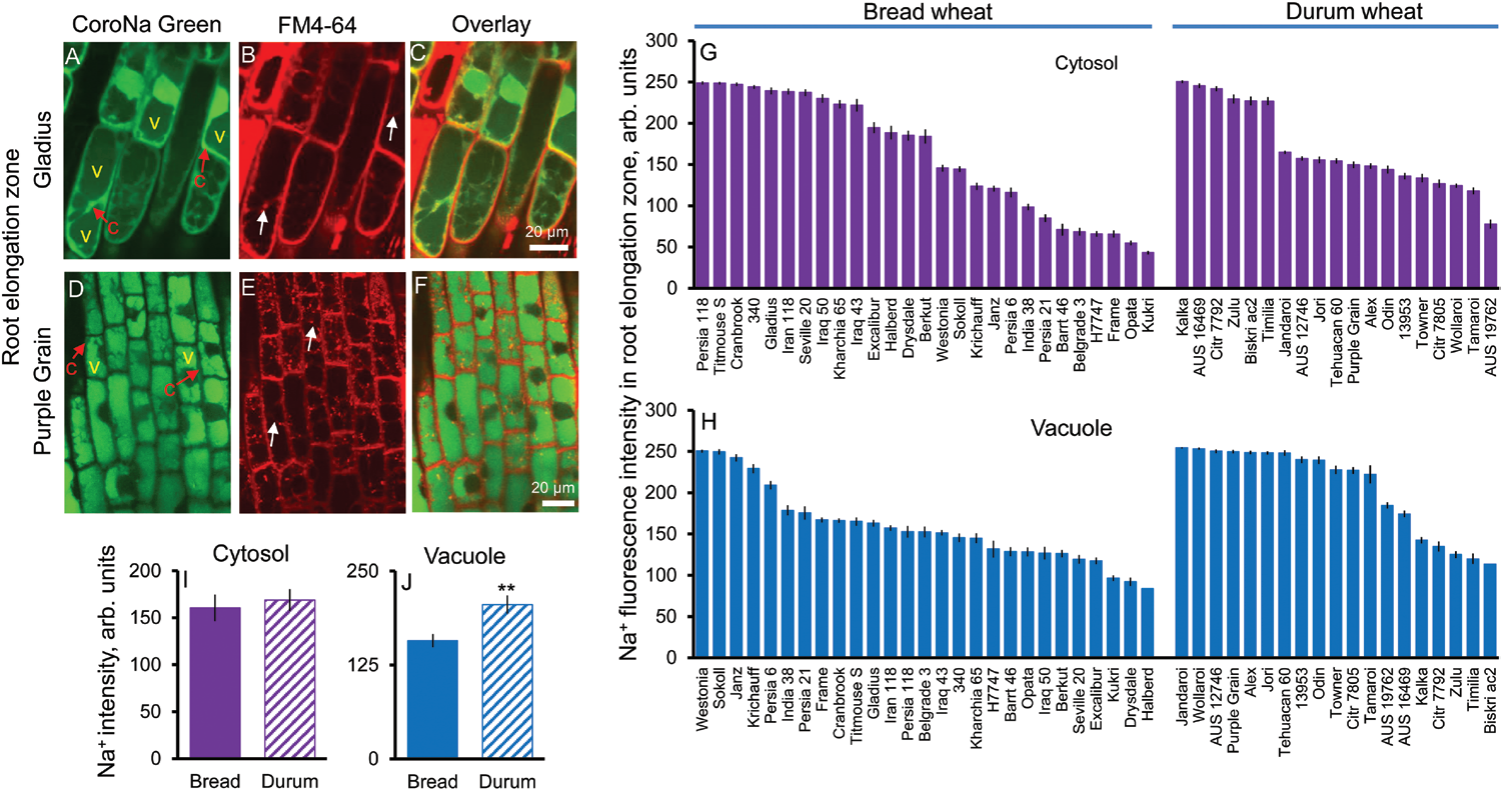
Genetic variability of Na^+^ intensity in the cytosol and vacuole in the root elongation zone. (A, B) and (D, E) Representative images of root elongation zone cells stained with CoroNa Green dye and FM4-64 membrane dye in bread wheat Gladius (A, B) and durum wheat Purple Grain (D, E) cultivars. (C and F) The corresponding overlay images. c, cytosol, v, vacuole. (G and H) Na^+^ content in the cytosolic (G) and vacuolar (H) compartments in the root elongation zone of 46 wheat varieties contrasting in their salinity tolerance quantified by the intensity of CoroNa Green fluorescence (arbitrary units). Mean ±SE (*n*=72–96 cells from at least six individual plants). (I and J) Averaged pooled values for Na^+^ intensity in the cytosol (I) and vacuole (J) in the root elongation zone for bread and durum wheat clusters. Mean ±SE [*n*=19 (durum wheat) and 27 (bread wheat)]. **Significant at *P*<0.01. Hydroponically grown 4-day-old wheat seedlings were treated with 100 mM NaCl for 72 h.

The profile of Na^+^ distribution between the cytosol and vacuole in the root elongation zone is shown in Fig. 2A–E. A large variability (5.8-fold) of cytosolic Na^+^ intensity was observed in all 46 wheat varieties tested, ranging from the highest value of 250 ± 2 (Kalka, durum wheat) to the lowest of 43 ± 2 (Kukri, bread wheat) (Fig. 2G). Similarly, a 3-fold variability was found for vacuolar Na^+^ intensity in the root elongation zone within the 46 screened wheat varieties (Fig. 2H). It ranged from the highest value of 254 ± 0.3 (Jandaroi, durum wheat) to the lowest of 83 ± 5 (Halberd, bread wheat) (Fig. 2H). No significant difference in cytosolic Na^+^ intensity in the root elongation zone was found between bread and durum wheat (mean fluorescence intensities 160 ± 14 and 169 ± 12, respectively; *P*>0.05; Fig. 2I). A significant negative correlation (*P*<0.05) was found between Na^+^ extrusion and cytosolic Na^+^ intensity in the root elongation zone (Supplementary Fig. S8A). At the same time, durum wheat showed an ~31% higher capacity for vacuolar Na^+^ sequestration (205 ± 12 and 157 ± 9, respectively; *P*<0.01; Fig. 2J) compared with bread wheat. A significant up-regulation (*P*<0.05) of *TaNHX1* in the root apex was found in durum wheat, whereas no change was found in bread wheat under salt stress (Supplementary Fig. S7C). Also, a significant negative correlation (*P*<0.05) was found between cytosolic and vacuolar Na^+^ intensity in the root elongation zone (Supplementary Fig. S8B). Taken together with the finding that bread wheat has significantly higher Na^+^ extrusion in the root elongation zone than durum wheat (Fig. 1B, C), our results suggest that both species are capable of maintaining the same level of cytosolic Na^+^ in the elongation zone, although by different mechanisms: in bread wheat, Na^+^ removal is directed towards the apoplast, while in durum wheat this is achieved by the higher extent of Na^+^ sequestration in the vacuole.

### Bread wheat possesses superior vacuolar Na^+^ sequestration ability in the root mature zone

**Fig. 3.**
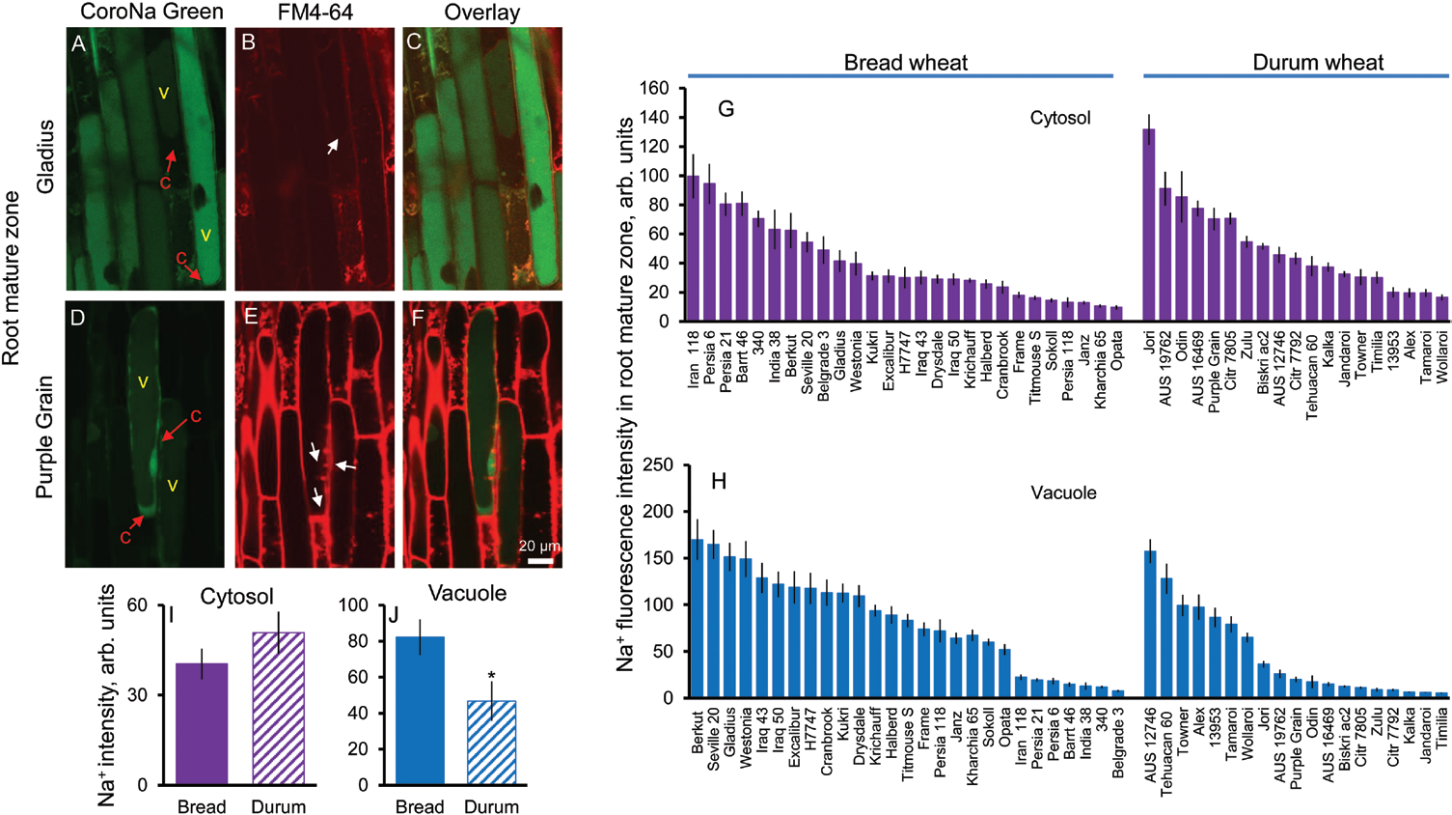
Genetic variability of Na^+^ intensity in the cytosol and vacuole in the root mature zone. (A, B) and (D, E) Representative images of root mature zone cells stained with CoroNa Green dye and FM4-64 membrane dye in bread wheat Gladius (A, B) and durum wheat Purple Grain (D, E) cultivars. (C and F) The corresponding overlay images. c, cytosol, v, vacuole. (G and H) Na^+^ content in the cytosolic (G) and vacuolar (H) compartments in the root mature zone of 46 wheat varieties contrasting in their salinity tolerance quantified by the intensity of CoroNa Green fluorescence (arbitrary units). Mean ±SE (*n*=72–96 cells from at least six individual plants). (I and J) Averaged pooled values for Na^+^ intensity in the cytosol (I) and vacuole (J) in the root mature zone for bread and durum wheat clusters. Mean ±SE [*n*=19 (durum wheat) and 27 (bread wheat)]. *Significant at *P*<0.05. Hydroponically grown 4-day-old wheat seedlings were treated with 100 mM NaCl for 72 h.

Given the rather limited volume of the root elongation zone, it is difficult to envisage that vacuolar Na^+^ sequestration in this zone will have a major impact on whole-plant Na^+^ relations and a control of its delivery to the shoot. Accordingly, we quantified profiles of the vacuolar and cytosolic Na^+^ distribution in the mature root zone (representing the major bulk of the root) (Fig. 3A–E). A large variability of Na^+^ intensity in the cytosol (13.6-fold, Fig. 3G) and the vacuole (31.4-fold, Fig. 3H) in the root mature zone was found in the screened varieties. Jori (durum wheat) showed the highest cytosolic Na^+^ intensity, 132 ± 10, whereas Opata (bread wheat) showed the lowest, 9.7 ± 1.4 (Fig. 3G). Furthermore, the highest vacuolar Na^+^ intensity was found in the variety Berkut (bread wheat), 170 ± 22, compared with the lowest, 5.4 ± 0.6, in the variety Timilia (durum wheat) (Fig. 3H). In the root mature zone, a significantly higher (75%) Na^+^ intensity in the vacuole was found in bread wheat than in durum wheat (82 ± 9 versus 47 ± 11, *P*<0.05; Fig. 3J), compared with no significant difference between bread and durum wheat in the cytosol (40.4 ± 5.1 versus 50.9 ± 7.0, *P*>0.05; Fig. 3I). A significant negative correlation (*P*<0.01) was found between cytosol and vacuolar Na^+^ intensity in the root mature zone (Supplementary Fig. S8C).

### Cytosolic Na^+^ accumulation in the root meristem zone was significantly higher in bread wheat

**Fig. 4.**
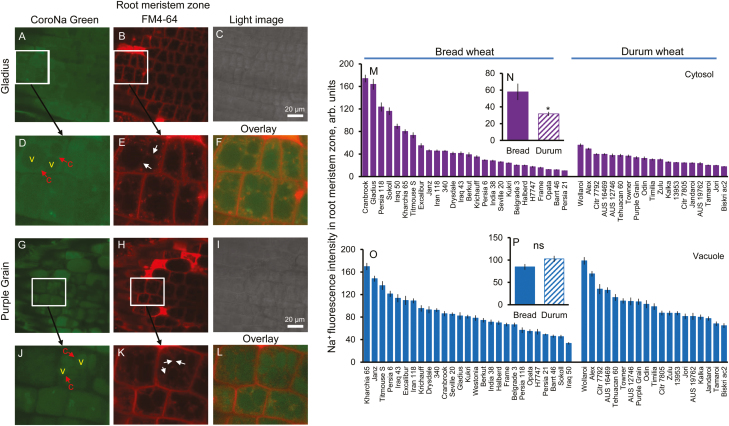
Genetic variability of Na^+^ intensity in the cytosol and vacuole in the root meristem zone. (A and G) Representative images of root meristem zone cells stained with CoroNa Green dye in bread wheat Gladius (A) and durum wheat Purple Grain (G) cultivars. (B, H) and (C, I) The corresponding FM4-64 tonoplast staining and light images, respectively. (D–F) and (J–L) The representative area of Na^+^ staining in Gladius and Purple Grain, respectively. c, cytosol, v, vacuole. (M and O) Na^+^ content in the cytosolic (M) and vacuolar (O) compartments in the root meristem zone of 46 wheat varieties contrasting in their salinity tolerance quantified by the intensity of CoroNa Green fluorescence (arbitrary units). Mean ±SE (*n*=72–96 cells from at least six individual plants). (N and P) Averaged pooled values for Na^+^ intensity in the cytosol (N) and vacuole (P) in the root meristem zone for bread and durum wheat clusters. Mean ±SE [*n*=19 (durum wheat) and 27 (bread wheat)]. *Significant at *P*<0.05. Hydroponically grown 4-day-old wheat seedlings were treated with 100 mM NaCl for 72 h.

To investigate further the intracellular Na^+^ distribution profiles in the root meristem, FM4-64 membrane dye was also used to co-stain wheat roots, allowing a clear visualization of Na^+^ distribution in various intracellular compartments. Representative images of Na^+^ distribution between the cytosol and vacuole (visualized by CornoNa Green dye and FM4-64 membrane dye) in the root meristem zone in bread (Gladius) and durum (Purple Grain) wheat are shown in Fig. 4A–L. A 16.7-fold variability in cytosolic Na^+^ intensity was observed within the 46 screened wheat varieties, ranging from the highest at 174 ± 7 (Cranbrook, bread wheat) to the lowest at 10.4 ± 0.4 (Persia 21, bread wheat) (Fig. 4M). Similarly, a large variability (5.4-fold) was found for vacuolar Na^+^ intensity in the root meristem zone, ranging from the highest value of 180 ± 7 (Wollaroi, durum wheat) to the lowest of 34 ± 2 (Iraq 50, bread wheat) (Fig. 4O). On average, a nearly 2-fold higher cytosolic Na^+^ intensity was found in bread wheat in this zone (57.9 ± 9.8 versus 31.7 ± 2.3, *P*<0.05; Fig. 4N). At the same time, no significant difference was found in vacuolar Na^+^ intensities between bread and durum wheat (85 ± 6 versus 103 ± 7, respectively, *P*>0.05; Fig. 4P). No significant (*P*>0.05) correlation was found between cytosol and vacuolar Na^+^ intensity in the root meristem zone Supplementary Fig. S8D).

### Tampering with the root meristem results in a salt-sensitive phenotype in bread wheat

**Fig. 5.**
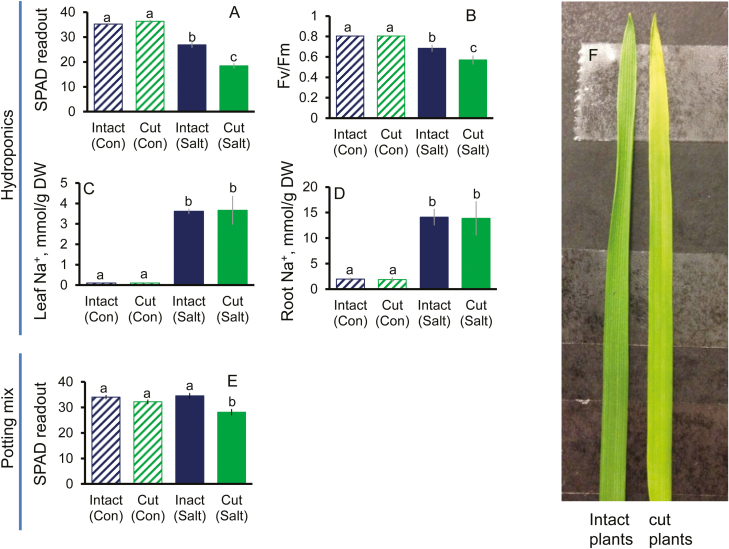
Comparative analysis of selected agronomical and physiological characteristics of plants after the surgical removal of the root meristem tissue. Salt-tolerant bread wheat variety Kharchia 65 was used. Plants were grown either hydroponically (A–D; 200 mM NaCl, 10 d) or in a potting mix (E; 200 mM NaCl, 15 d) and treated with 200 mM NaCl for either 10 d or 15 d, respectively. Chlorophyll content (SPAD value) (A for hydroponic experiment, E for potting mix experiment), maximum photochemical efficiency of PSII (chlorophyll fluorescence *F*_v_/*F*_m_ value, arbitrary units) (B), and leaf (C) and root (D) Na^+^ content were measured at the end of the experiment and compared between intact (with intact roots) and cut plants (in which root meristems were removed). Mean ±SE (*n*=24−36). (F) The comparison between leaves from intact and cut hydroponically grown plants after 10 d of salt stress. Different lower case letters denote significant difference between data at *P*<0.05. Con, non-saline condition.

From the above data, we have hypothesized that the root meristem may operate as, or harbour, a salt stress sensor. According to this hypothesis, a stress-induced increase in the cytosolic Na^+^ in this zone may be essential to induce a cascade of signalling and adaptive responses, at both the tissue and the whole-plant level. To test this hypothesis, experiments were conducted using the salt-tolerant Kharchia 65 bread wheat variety in an attempt to modify salinity stress tolerance by tampering with salt sensing in root meristems. Under control conditions, the surgical removal of the meristematic tissue in seminal roots did not affect whole-plant physiological characteristics (Fig. 5A–D), as judged by leaf chlorophyll content (SPAD readings; Fig. 5A), maximal photochemical efficiency of PSII (Fig. 5B), and leaf (Fig. 5C) and root (Fig. 5D) Na^+^ content. At the same time, the same procedure has resulted in a salt-sensitive phenotype under stress conditions, with significantly higher SPAD (Fig. 5A, F) and *F*_v_/*F*_m_ (Fig. 5B) values found in intact plants (with intact roots) compared with cut plants (with removal of the root meristem zone) in hydroponic experiments. Interestingly, no difference in the total Na^+^ content in either leaf or root was found between intact plants and those which had been ‘tampered with’ (Fig. 5C, D). This may be indicative that while the overall rate of Na^+^ uptake and transport to the shoot was not altered by the removal of the root meristem, the distribution of Na^+^ between the cytosol and vacuole in mesophyll cells might be different between the intact plants and cut plants. Needless to say, detrimental effects of salinity are not necessarily determined by the absolute amounts of salt accumulated in the shoot but rather its distribution between the intracellular compartments, as shown in numerous studies on halophytes.

**Fig. 6.**
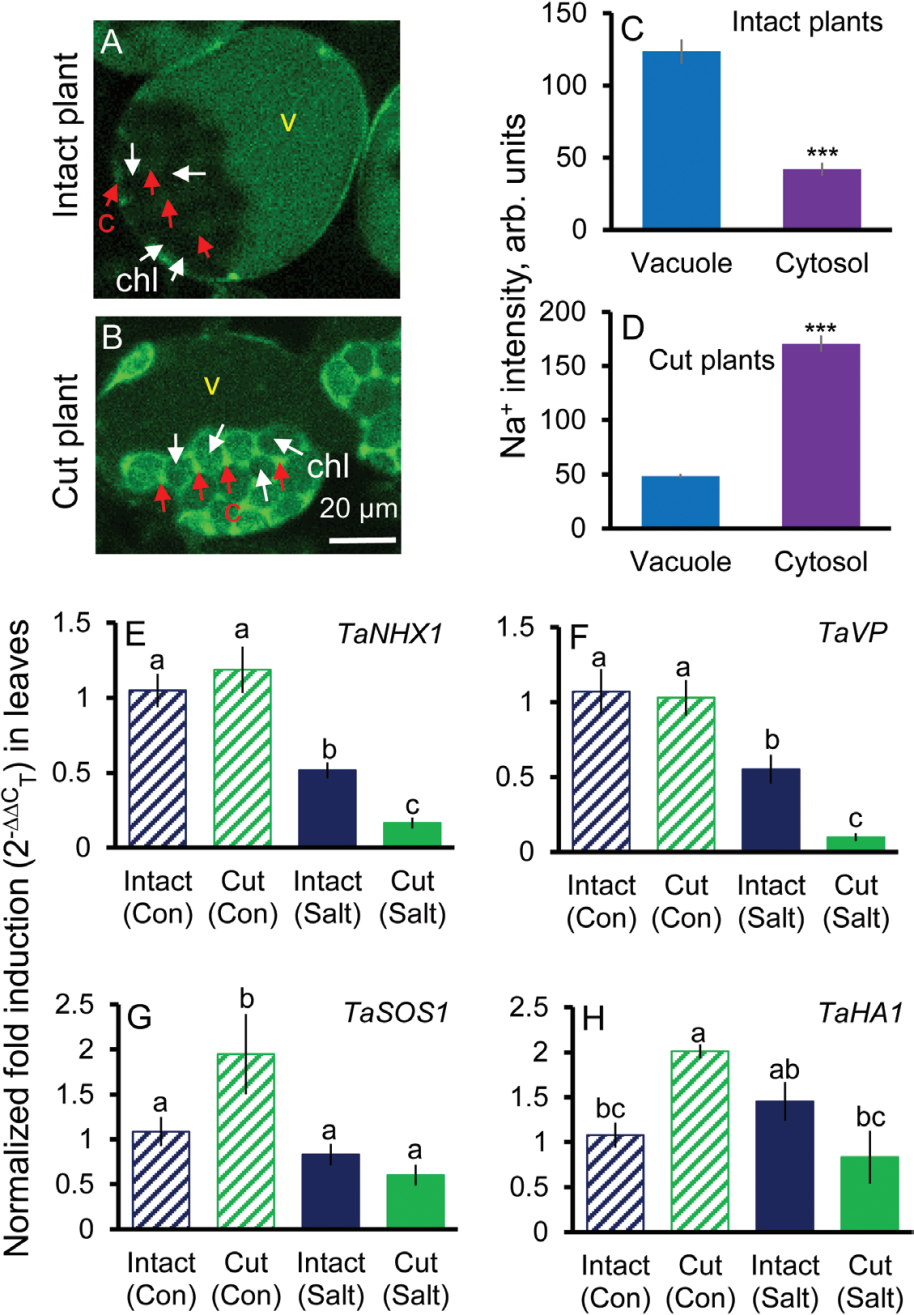
Na^+^ distribution and relative expression levels of several key genes related to Na^+^ transport in leaf mesophyll cells in intact plants and plants with the root meristems removed. Representative images showing CoroNa Green dye signal from leaf mesophyll cells in intact plants (with intact roots) (A) and cut plants (in which the root meristem tissues were removed) (B). (C and D) Intensity of CoroNa Green fluorescence (arbitrary units) in the cytosol and vacuole in the intact and cut plants, respectively. Mean ±SE (*n*=47−86 cells from at least three individual plants). c, cytosol; v, vacuole; chl, chloroplast. Plants were grown in potting mix under 200 mM NaCl for 15 d. (E– H) Comparison of transcriptional profiles of genes mediating Na^+^ removal from the cytosol [*TaNHX1* (E), *TaVP* (F), *TaSOS1* (G), and *TaHA1* (H)] in the youngest fully expanded leaves of intact plants and plants in which root meristems were surgically removed. Plants were grown hydroponically in the presence of 200 mM NaCl for 10 d. Mean ±SE (*n*=3 biological replicates). The salt-tolerant bread wheat variety Kharchia 65 was used in experiments. ***Significant at *P*<0.001. Different lower case letters mean *P*<0.05.

To confirm the above hypothesis, we repeated the same experiment in pot-grown (rather than hydroponically grown) plants exposed to more prolonged (15 d) salt stress. Similar to the results in the hydroponic experiment, significantly higher SPAD values were found in intact plants than in cut plants grown under salt stress conditions (Fig. 5E) but not in non-saline conditions. Staining mesophyll cells with CoroNa Green dye revealed that cells from intact plants possessed a 3- to 4- fold higher ability to remove Na^+^ from the cytosol and sequester it into the mesophyll cell vacuoles, compared with plants in which root meristems were removed (Fig. 6A–D). These might cause both higher Na^+^ toxicity and lower ability for osmotic adjustment executed by the vacuolar Na^+^, and thus result in higher sensitivity to salt stress in the plants which were tampered with. Consistent results demonstrating the salt-sensitive phenotype caused by the removal of the root meristems were also reported in experiments involving other bread and durum wheat genotypes and different durations and levels of saline stress (Supplementary Figs S9, S10). Taken together, these data strongly support the notion that tampering with root salt sensing by the meristem has major consequences for the long-distance stress signalling between the root and shoot and vacuolar Na^+^ sequestration in the shoot.

### Tampering with the root meristem affects transcription of genes conferring Na^+^ sequestration in the vacuole of mesophyll cells

We aimed to reveal if the above effect of root meristem stress sensing on Na^+^ sequestration in the vacuole of mesophyll cells was due to transcriptional or post-translational regulation. For this, we have compared transcriptional profiles of several key genes potentially mediating the above process. Theu included the *TaNHX1* gene encoding the tonoplast Na^+^(K^+^)/H^+^ exchanger; genes for two tonoplast-based H^+^-pumps fuelling NHX1 activity (*TaHA1* for H^+^-ATPase and *TaVP* for H^+^-PPase); and the *TaSOS1* gene encoding the plasma membrane Na^+^/H^+^ antiporter. In both intact plants and those that were ‘tampered with’, salinity stress reduced transcript levels of *TaNHX1* and *TaVP* (Fig. 6E, F), but in plants deprived of the ability to sense salt stress by the root meristem the effect was much more (2- to 3-fold stronger) dramatic. The transcript levels of *TaHA1* and *TaSOS1* were not affected by salinity in intact plants but were reduced 2- to 3-fold in plants with root meristems removed (Fig. 6G, H). Interestingly, transcript levels of *TaSOS1* and *TaHA1* were significantly higher in cut plants than in intact plants under non-saline condition (Fig. 6G, H).

## Discussion

### Na^+^ extrusion from the root elongation zone is essential for salt tolerance in wheat

Most authors have a tendency to use the term ‘sodium exclusion’ in a very broad sense, and often as a synonym for prevention of its accumulation in the shoot. As a result, the actual role of Na^+^ extrusion from the root, and its contribution to the differential salt tolerance between bread and durum wheat remains largely unknown. A comparison was done by monitoring ^22^Na^+^ uptake in radiotracer experiments (Davenport *et al.*, 1997; Z. Chen *et al.*, 2007); neither of these experiments has revealed any significant correlations between plant salinity tolerance and unidirectional Na^+^ uptake by roots, with all studied wheat and barley cultivars showing more or less the same rate of ^22^Na^+^ uptake.

Na^+^/K^+^-ATPase expels three Na^+^ ions from the cell in exchange for two K^+^ ions in animal cells (Yatime *et al.*, 2011; Galva *et al.*, 2012). In lower plants, such as *Physcomitrella patens*, extrusion of Na^+^ can be achieved by sodium ATPase (PpENA1) (Benito and Rodríguez-Navarro, 2003; Lunde *et al.*, 2007). In higher plants, however, the SOS1 Na^+^/H^+^ antiporter remains the only known transporter to exclude Na^+^ from the cytosol into the apoplast. β-Glucuronidase (GUS) expression analysis showed that SOS1 was preferentially expressed in the root apex but not the mature zone in Arabidopsis (Shi *et al.*, 2002). This was confirmed by our functional studies on wheat. While in the mature root zone Na^+^ efflux mostly ranged between –20 nmol m^–2^ s^–1^ and –70 nmol m^–2^ s^–1^ (Cuin *et al.*, 2011), 10- to 20-fold higher Na^+^ extrusion ability was observed in this study (for the same conditions) in the root elongation zone, with net Na^+^ fluxes ranging from –284 ± 39 nmol m^–2^ s^–1^ to –1584 ± 237 nmol m^–2^ s^–1^ (Fig. 1). These Na^+^ fluxes measured by the MIFE Na^+^-selective microelectrodes were sensitive (>80% inhibition; Supplementary Fig. S2) to amiloride, a known blocker of the mammalian Na^+^/H^+^ exchanger (Kleyman and Cragoe, 1988) and were not observed in an Arabidopsis mutant lacking a functional *SOS1* gene (Supplemenatary Fig. S2). As a known mammalian Na^+^/H^+^ exchanger blocker, amiloride is also widely used in different plant species (Blumwald *et al.*, 1987; Fukuda *et al.*, 1998; Qiu *et al.*, 2003; Pardo *et al.*, 2006; Cuin *et al.*, 2011). Taken together, this implies that Na^+^ fluxes reported in this work were mediated mainly by SOS1-like plasma membrane Na^+^/H^+^ exchangers in epidermal cells in the wheat root elongation zone. Thus, the reported finding of significantly higher (by 33%; *P*<0.05; Fig. 1) Na^+^ extrusion ability of bread wheat measured by MIFE provides the first functional evidence for the role of the SOS1 extrusion mechanism as the factor conferring differential salt stress tolerance between bread and durum wheat species, as all the reported evidence to date has attributed this trait to shoot tissue (Cuin *et al.*, 2010; Munns *et al.*, 2012). Although the bulk of the root is comprised of mature root cells, the possibility of internal Na^+^ transport within the root cortex is highly likely. Thus, the scenario where Na^+^ taken up by the bulk of the root is trafficked to the apex and then extruded via SOS1 exchangers cannot be ruled out.

### Root vacuolar Na^+^ sequestration in the mature zone explains superior bread wheat performance under saline conditions

While vacuolar Na^+^ sequestration is unequivocally accepted as a key trait conferring salinity tissue tolerance, the absolute majority of published papers referred to this mechanism operating in the shoot, both in halophytes (Flowers and Colmer, 2008; Shabala and Mackay, 2011) and in glycophytes (Apse *et al.*, 1999; Munns and Tester, 2008). In this work, we provide the first large-scale evidence that such a trait is also applicable to plant roots and is essential to confer the difference in salinity stress tolerance between related species.

The physiological rationale behind this phenomenon may be the fact that the mature root zone represents the major bulk of the root and thus possesses enough capacity to reduce the Na^+^ load to the shoot. HKT-mediated retrieval of Na^+^ from the xylem has been widely reported to be linked to reduced Na^+^ accumulation in the shoot (Sunarpi *et al.*, 2005; Huang *et al.*, 2006; Byrt *et al.*, 2007) and ultimately salt tolerance in wheat (Munns *et al.*, 2012). However, there is not much physiological rationale to load Na^+^ actively in the shoot (see Shabala, 2013 for thermodynamic analysis and arguments) and then retrieve it back. This futile cycle can be avoided, and the same effect can be achieved at a lower cost by immediate deposition of salt load in the root cortex in the mature zone. More interestingly, vacuolar fluorescence Na^+^ signal intensity is much higher in the root elongation zone than in the mature zone in wheat (Figs 2, 3), suggesting that Na^+^ might be trafficked from the root mature zone to the elongation zone. This is in agreement with the finding that the root apex has a significantly higher sap Na^+^ content than the mature root tissue (Supplementary Fig. S11).

The above pattern and correlation between vacuolar Na^+^ concentration in root cells and overall plant salinity stress tolerance does not hold for the elongation zone (Fig. 2). Here, durum wheat compensates poor Na^+^ extrusion ability from the elongation zone by more efficient vacuolar Na^+^ sequestration. The ultimate result is the same: both species maintain the same Na^+^ concentrations in the cytosol in the root elongation zone, though by different mechanisms. It may also be tempting to suggest that this strategy may allow durum wheat to adjust osmotically and maintain cell elongation by mainly relying on (freely available) Na^+^. The energy cost of osmotic adjustment by means of inorganic ions is an order of magnitude lower than by *de novo* synthesis of compatible solutes (Shabala and Shabala, 2011; Munns and Gilliham, 2015). It also should be noted that a possible contribution of accumulation of compatible solutes in the cytoplasm to equilibrate increased vacuolar osmolality cannot be fully ruled out, especially in the elongation and mature root cells which have a central vacuole. As durum wheat possesses a much lower root K^+^ retention ability compared with bread wheat (Cuin *et al.*, 2008), higher reliance of Na^+^ may be suggested as a plausible strategy to achieve osmotic adjustment without redirecting additional ATP resources for organic osmolyte biosynthesis.

### Na^+^ sequestration in mesophyll cells was affected by the removal of the root meristem tissue

Plant adaptive responses to the environment require orchestrated regulation of a plethora of physiological mechanisms and thus imply efficient root to shoot communication (S. Shabala *et al.*, 2016). The communication modes between the root and shoot are highly diverse and include a broad range of physical [electric and hydraulic signals, propagating Ca^2+^ and reactive oxygen species (ROS) waves], chemical (assimilates, hormones, and nutrients), and other molecular (peptides, proteins, and RNA) signals (Baxter *et al.*, 2014; Gilroy *et al.*, 2014; Van Bel *et al.*, 2014; S. Shabala *et al.*, 2016). Any of these signals may potentially be responsible for the observed salt-sensitive phenotype after tampering with the root meristem signalling.

**Fig. 7.**
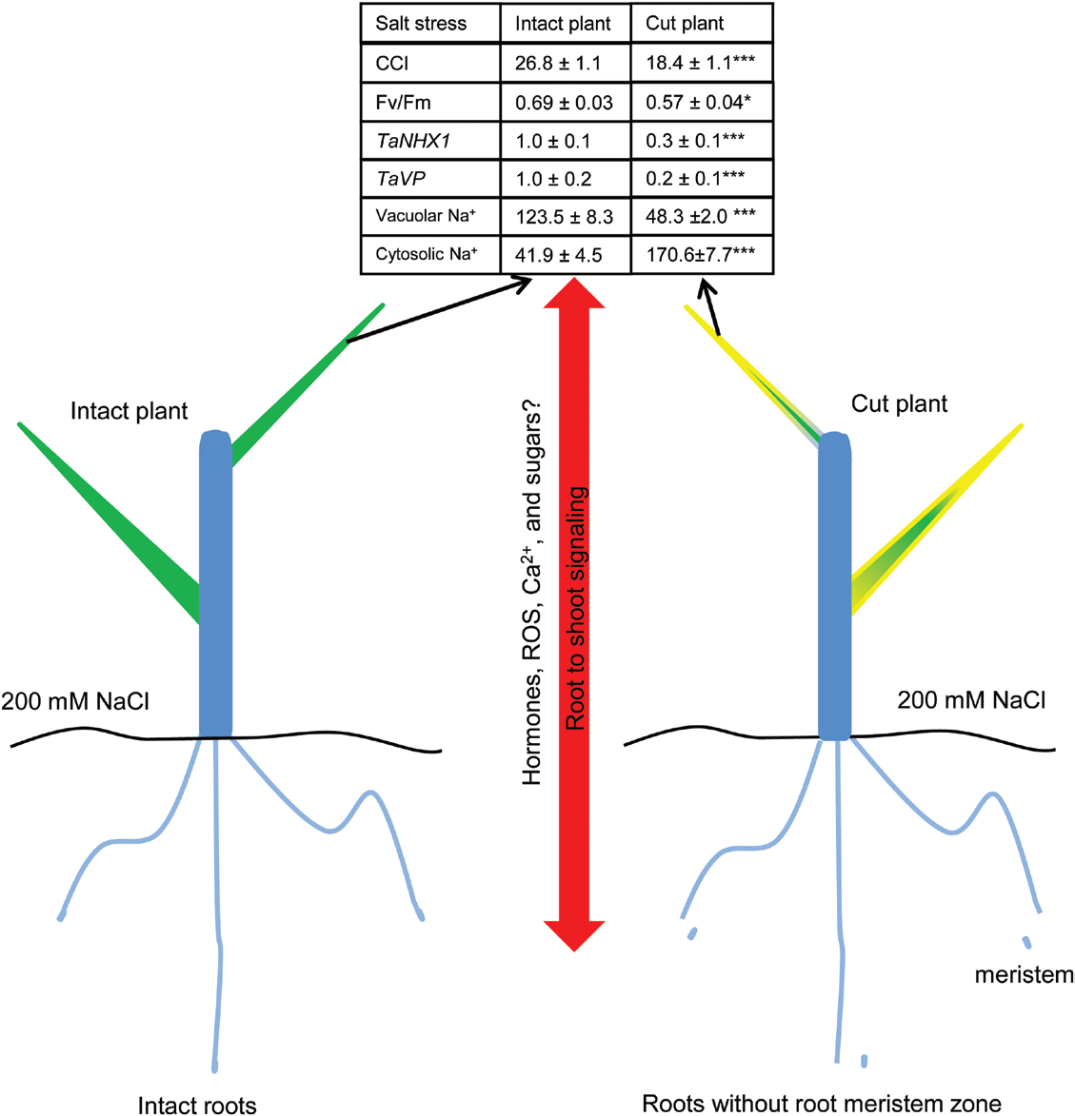
The proposed model comparing major changes in gene expression profiles, Na^+^ ion distribution, and phenotyping responses in leaves of plants compromised in salt sensing by the meristem tissue. Data in the table are significant at *P<0.05 and ***P<0.001.

Our current knowledge of how salt stress is sensed by plant tissues is severely limited and, while several mechanisms have been considered as suitable to undertake the role of the putative salt sensor (for the most recent reviews, see Maathuis, 2014; Shabala *et al.*, 2015), no explicit evidence has been provided in the literature. This work makes an important contribution to filling this knowledge gap. We first provide strong evidence that such a salt stress sensor is located in the root meristem. Indeed, removing the meristem tissue resulted in a salt-sensitive phenotype (Figs 5, 7; Supplementary Fig. S9) in otherwise highly salt-tolerant Kharchia 65 and Westonia wheat varieties. Secondly, we showed that meristem signalling to the shoot is essential in enabling vacuolar Na^+^ sequestration ability in leaf mesophyll cells, in a process that is at least partially conferred by differential expression of a set of Na^+^ transport-related genes at the tonoplast (Fig. 6). Thirdly, given the fact that both durum and bread wheat had experienced the same osmotic stress (and hence tension on the plasma membrane), our results rule out mechanosensing mechanisms (Monshausen and Haswell, 2013) as a key determinant of differential salinity stress tolerance between durum and bread wheat. Instead, taken together, our results point out the important role of elevated cytosolic Na^+^ in the signalling process, narrowing down the list of candidate mechanism to only a few. The first candidate may include the SOS1 Na^+^/H^+^ antiporter *per se* operating in the sensing mode (Zhu, 2003). This protein has a very long tail (700 amino acids) residing in the cytosol that potentially senses Na^+^. The second Na^+^ sensor candidate in systems may refer to the proteins which comprise the side chains of an aspartate and a histidine that form a DxR/KxxH motif (Thomson *et al.*, 2015), and Maathuis (2014) has estimated that ~1200 candidate proteins containing this motif exist in Arabidopsis. Future studies therefore should focus on tissue-specific profiles of expression of some of these candidate genes. The third potential candidates are NCX Na^+^/Ca^2+^ exchangers (Shabala *et al.*, 2015). A recent bioinformatics analysis (Wang *et al.*, 2012) suggests the existence of a putative NCX gene in Arabidopsis, which shows increased transcript levels when plants are exposed to salinity.

Although recent findings emphasized the essential role of AtNHX1 in K^+^ accumulation in the vacuole (Bassil *et al.*, 2011; Barragán *et al.* ,2012), they do not completely disregard its involvement in vacuolar Na^+^ sequestration, and it is highly plausible that NHX1 has a dual role (Na^+^ and K^+^/H^+^ antiporter) (Maathuis, 2014; Liu *et al.*, 2017). While the tonoplast NHX1 Na^+^(K^+^)/H^+^ exchanger is critical for vacuolar Na^+^ sequestration and thus plant salt tolerance (Apse *et al.*, 1999), for its operation it should be fuelled by either vacuolar PPase or H^+^-ATPase (Beyenbach and Wieczorek, 2006; Silva and Gerós, 2009). Here we showed that preventing meristem-mediated root to shoot salt signalling resulted in a significant down-regulation of transcript levels in both tonoplast pumps (Fig. 6). This had a profound effect on vacuolar Na^+^ sequestration (Fig. 6) and overall plant performance under saline conditions. Salt stress-induced nuclear and DNA degradation and programmed cell death phenotypes were also found in root meristematic cells (Liu *et al.*, 2000; Huh *et al.*, 2002). Moreover, under salt stress, the NHX1 gene is up-regulated in Arabidopsis (Gaxiola *et al.*, 1999). However, the story in wheat might be different (Fig. 6E). Mullan *et al.* (2007) showed that the transcript level of NHX1 in wheat was almost unchanged in the fourth leaf blade under 200 mM NaCl, whereas a clear decrease was observed in roots. Thus, the tissue-specific aspect should be accounted for. Other factors such as treatment time, leaf age, and salt stress concentration should also be considered.

Revealing the molecular nature of the root to shoot signal controlling vacuolar Na^+^ sequestration in the mesophyll (and ultimately salinity tissue tolerance) remains a challenging but highly important task for the future. Importantly, different signalling systems operate at very different time scales, and it was also suggested that some of these systems may operate in a priming mode(s), while others deliver more specific information about the precise nature of the signal (S. Shabala *et al.*, 2016). So, what is the nature of the salt-induced root signals?

It is known that the cytokinin and auxin antagonistic interaction is important in controlling meristem activity (Del Ioio *et al.*, 2007, 2008). Thus, the change at the hormonal level might be one explanation for the altered Na^+^ distribution in salinized plants after removal of the roots meristem zone. Other candidates such as brassinosteroid (Hacham *et al.*, 2011), ROS (Gilroy *et al.*, 2014), Ca^2+^ (Dodd *et al.*, 2010), abscisic acid (Liang *et al.*, 2007), and sugars (Rosa *et al.*, 2009; Krasensky and Jonak, 2012) may participate in the long-distance salt stress signal transduction and thus should be considered in future work (Fig. 7). ROS were previously suggested to play a role in systematic signal transduction under abiotic stress (Miller *et al.*, 2009; Mittler *et al.*, 2011; Baxter *et al.*, 2014). Ca^2+^ is known as a second messenger and is involved in a broad range of plant cell activities in regulating plant growth and development (Hepler, 2005). Recently, salt stress-induced Ca^2+^ waves were found to be associated with rapid, long-distance root to shoot signalling in plants (Choi *et al.*, 2014). Furthermore, overexpression of ABF2 (ABRE-binding bZIP factor), an essential component of glucose signalling, improved salt stress tolerance in Arabidopsis (Kim *et al.*, 2004). Taken together, the signalling events involved in this phenomenon may be a complex co-ordinated by different signalling molecules in a ‘fine tune’ and/or ‘coarse tune’ mode. Future studies should be conducted to investigate the identity of specific signalling molecules and their role in the phenomenon whereby leaf Na^+^ distribution in salinized plants was affected by the removal of its root meristem tissue.

## Supplementary data

Supplementary data are available at *JXB* online.

Fig. S1. Genetic variability in salinity stress tolerance (quantified as a tolerance index) amongst 46 wheat varieties and its correlation with relative grain yield under salt stress.

Fig. S2. Na^+^ efflux measured by the MIFE technique from plant roots using the ‘recovery protocol’ is mediated by SOS1 Na^+^/H^+^ exchangers.

Fig. S3. Quantification of intracellular Na^+^ distribution in the cytosol and vacuole.

Fig. S4. The effect of CoroNa Green dye concentration on the intensity of the fluorescent Na^+^ signal in wheat roots measured from different root zones.

Fig. S5. The effect of incubation time of CoroNa Green (20 µM) on the intensity of the fluorescent Na^+^ signal in wheat roots measured from different root zones.

Fig. S6. The procedure for removal of the root meristem tissue.

Fig. S7. The relative gene expression patterns in the root apex and mature root in bread (cv. Gladius) and durum wheat (cv. Purple Grain) after salt stress.

Fig. S8. Correlations between cytosolic and vacuolar Na^+^ intensity and Na^+^ efflux in wheat cultivars.

Fig. S9. Removal of the root meristem results in a salt-sensitive phenotype.

Fig. S10. Removal of the root meristem results in higher vulnerability of durum wheat (cv. Wallaroi) to salt stress.

Fig. S11. Sap Na^+^ content in the root apical and mature tissues.

Table S1. Primers used in this study.

Supplementary Material

## Abbreviations

ABF2: ABRE-binding bZIP factor
HKT: high affinity K^+^ transporter
LIX: liquid ion exchanger
LSCM: laser scanning confocal microscopy
MIFE: microelectrode ion flux estimation
NCX: Na^+^/Ca^2+^ exchanger
ROS: reactive oxygen species
SOS1: Salt Overly Sensitive 1
TaHA1: bread wheat H^+^-ATPase
TaVP: bread wheat H^+^-PPase
