# Supplementary material for: Na+ extrusion from the cytosol and tissue-specific Na+ sequestration in roots confer differential salt stress tolerance between durum and bread wheat
Source: J Exp Bot. 2018 Jun 11;69(16):3987–4001. doi: 10.1093/jxb/ery194 (PMC6054258; doi:10.1093/jxb/ery194)
Supplement: Supplementary Material [file ery194_suppl_supplementary_materials.pdf]

Na^+ extrusion and tissue-specific Na^+ sequestration in roots confer differential salt stress tolerance between durum and bread wheat

Honghong Wu[1,4], Lana Shabala[1], Elisa Azzarello[2], Yuqing Huang[3], Camilla Pandolfi[2], Nana Su[1], Qi Wu[1], Shengguan Cai[3], Nadia Bazihizina[1], **XXX** Lu[1], Meixue Zhou[1], Stefano Mancuso[2], Zhonghua Chen[3], Sergey Shabala[1*]

[1], School of Land and Food, University of Tasmania, Private Bag 54, Hobart, Tasmania 7001, Australia

[2], Department of Horticulture, University of Florence, 50019, Sesto Fiorentino, Italy

[3], School of Science and Health, Hawkesbury Institute for the Environment, Western Sydney University, Penrith, NSW 2751, Australia

[4], Current address: Department of Botany and Plant Sciences, University of California, Riverside, California 92521, USA

*****, Address correspondence to Sergey.Shabala@utas.edu.au

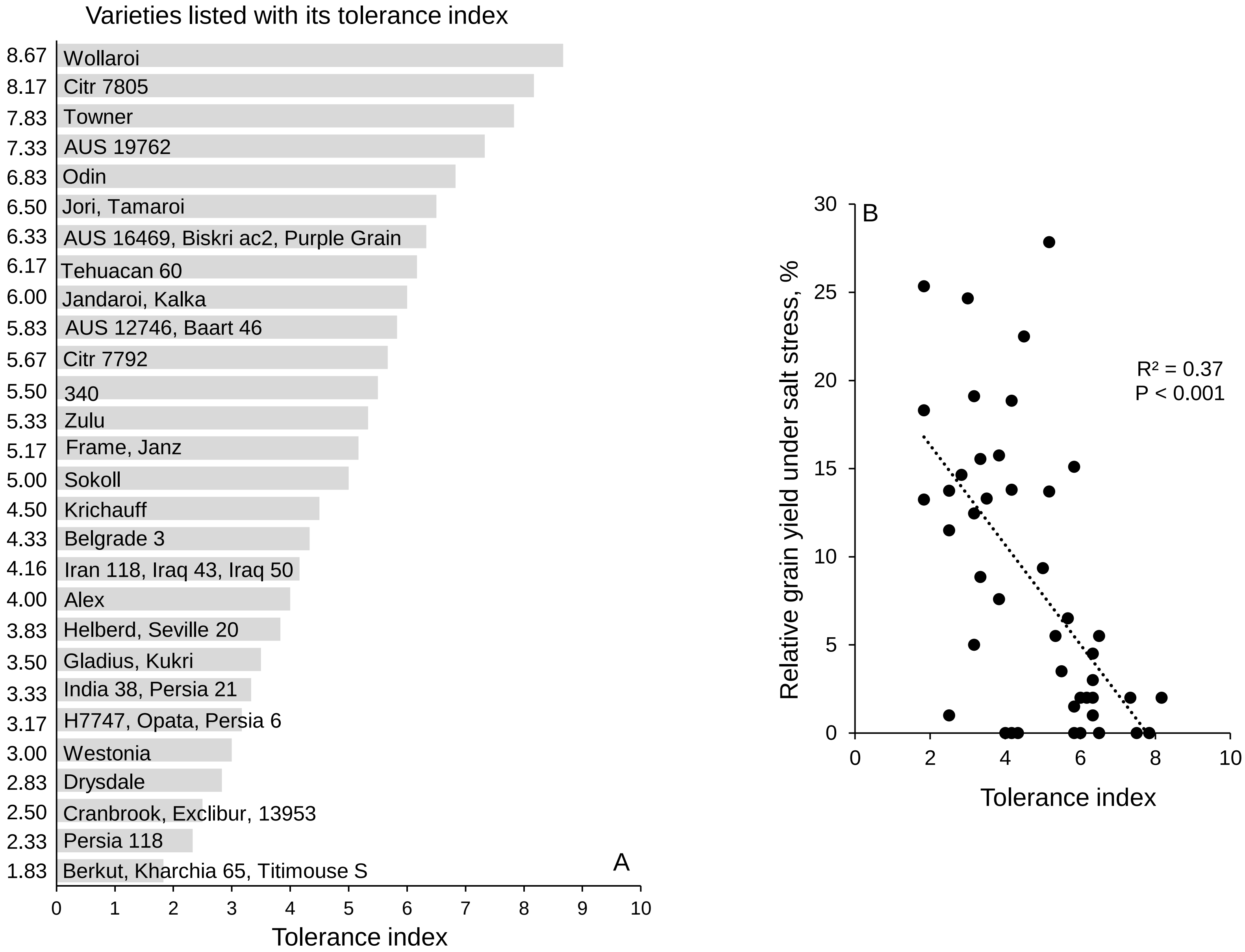

Figure S1. Genetic variability in salinity stress tolerance (quantified as a tolerance index) amongst 46 wheat varieties (A) and its correlation with relative grain yield (B) under salt stress. Each point in panel (B) represents a separate variety. Based on Wu et al., 2014. Plants were grown under glasshouse conditions and treated with 300 mM NaCl for 6 weeks.

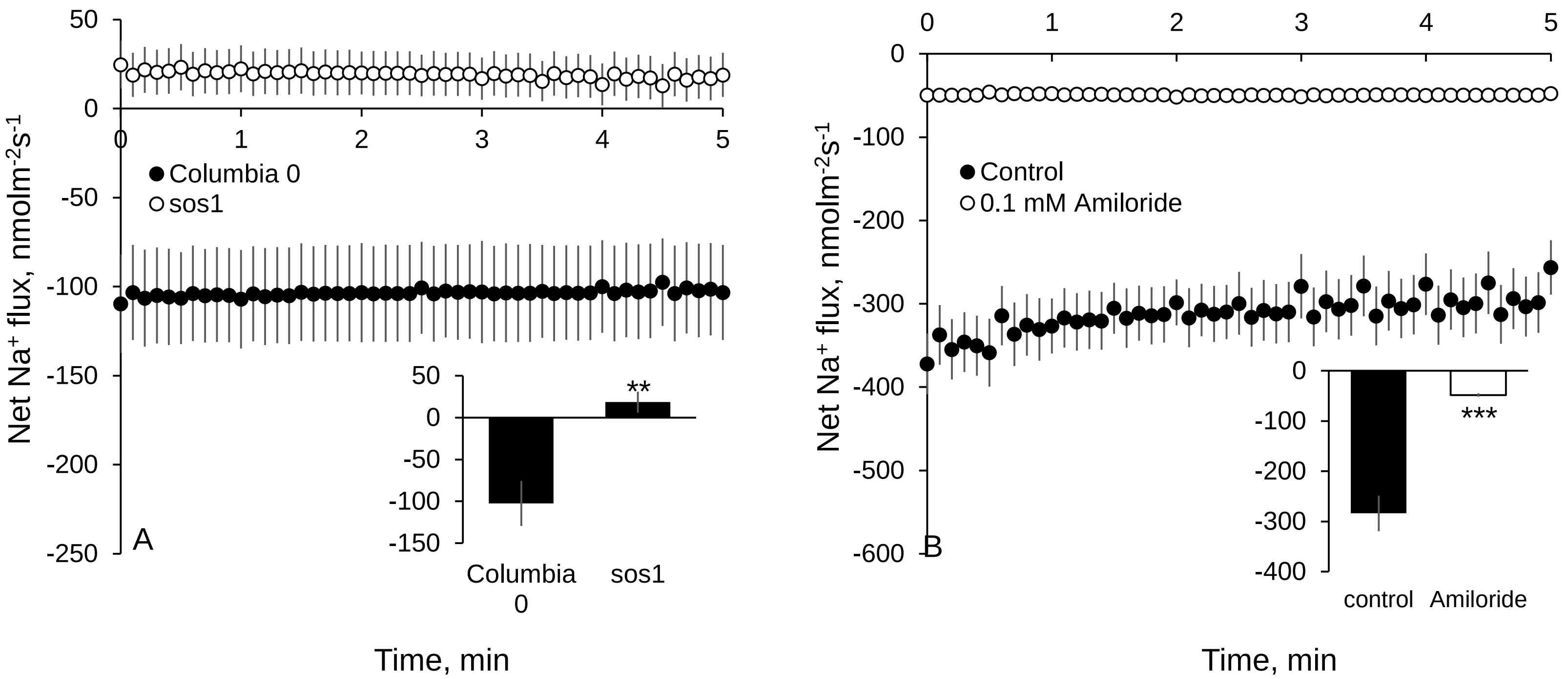

Figure S2. Na^+ efflux measured by microelectrode MIFE technique from plant roots using "recovery protocol" is mediated by SOS1 Na^+/H^+ exchangers. Data adapted from Cuin et al., 2011. A, Steady-state net Na^+ efflux measured 15 min after NaCl removal from roots of *Arabidopsis* wild type Columbia 0 and *sos1* mutants. Measurements were conducted from the root elongation zone (0.1 mm from the root tip) from 6-d-old plants after treating them with 150 mM NaCl for 24 h and then transferring them to Na-free solution. Mean ± SE (n = 6 to 8). Plants lacking functional SOS1 exchangers are not capable to actively extrude Na^+ while this capacity is present in a wild type line. B, Steady-state net Na^+ efflux measured in recovery protocol (as above) are sensitive to amiloride, a known inhibitor of Na^+/H^+ exchanger. Measurements were conducted on roots of bread wheat Kharchia 65 after removal of 150 mM NaCl (24h treatment) from root pre-incubated with 0.1 mM amiloride for 1 h. Mean ± SE (n = 6 seedlings). Significant at ** $P < 0.01$ and *** $P < 0.001$.

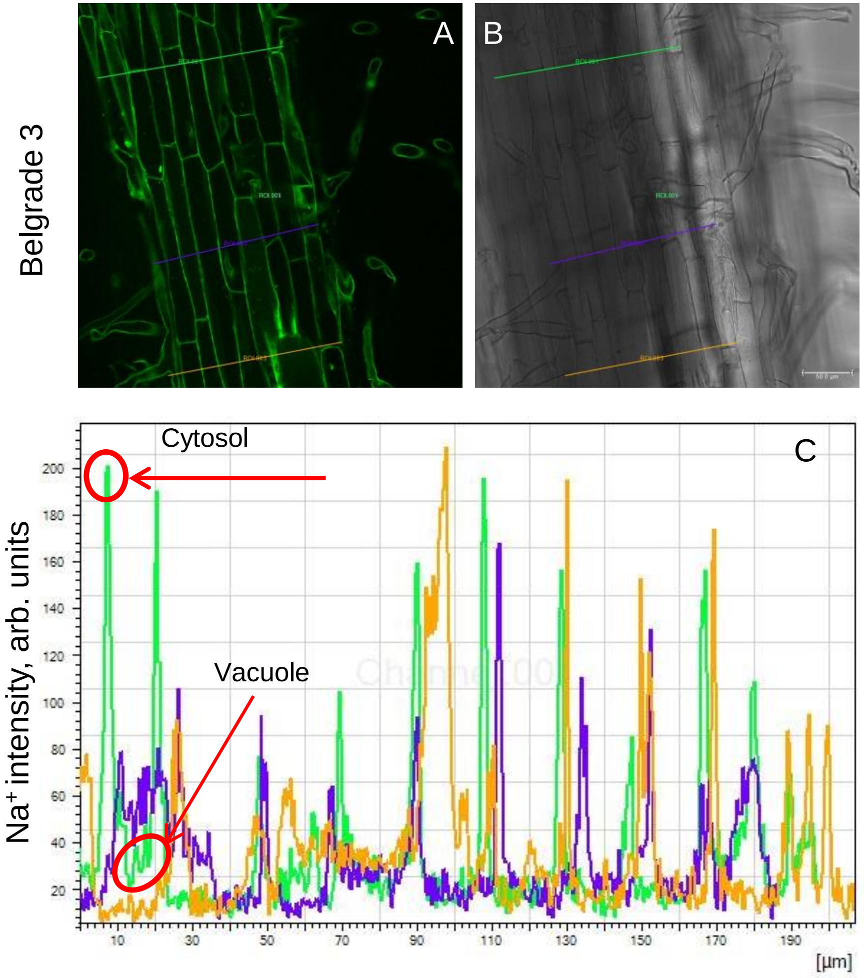

Figure S3. Quantification of intracellular Na^+ distribution in the cytosol and vacuole (illustrated by using one typical image of the mature root zone of bread wheat variety Belgrade 3). Hydroponically grown 4 days old wheat seedlings were treated with 100 mM NaCl for 72h. A, Several lines are drawn across the so-called "region of interest" (ROI) in an appropriate root zone. B, The corresponding light images. C, Continuous fluorescence intensity distribution profiles obtained by LAS AF Lite software. The mean fluorescence intensity values for the cytosol and vacuole are then calculated for each cell by attributing signal profiles to root morphology (visualised by light microscopy images; not shown in this figure).

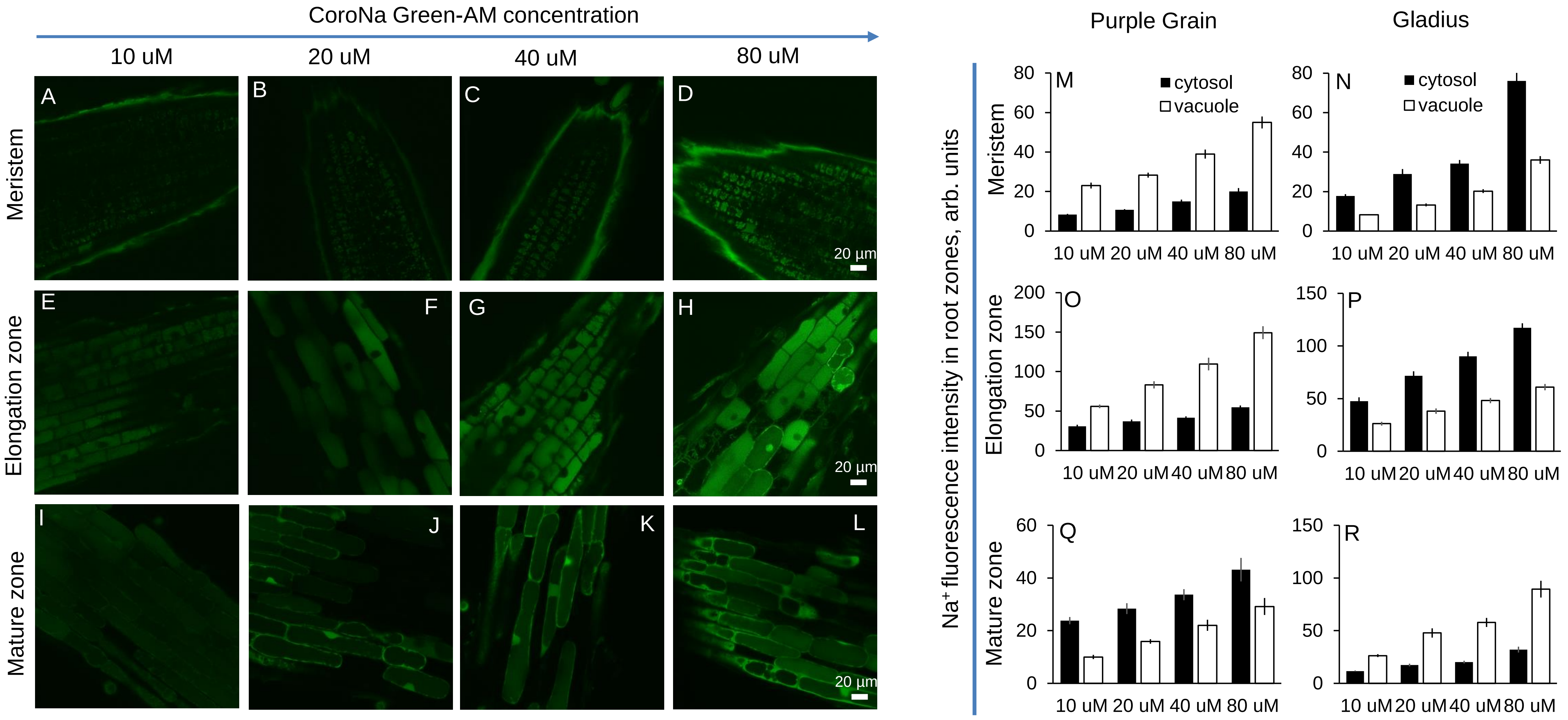

Figure S4. The effect of CoroNa Green dye concentration on the intensity of fluorescent Na^+ signal in wheat roots measured from different root zones. Hydroponically grown 4 days old wheat seedlings were treated with 100 mM NaCl for 72h. A-L, Representative (one of 4) confocal images of wheat variety (cv. Purple Grain) are shown for each zone for roots loaded with 10, 20, 40, 80 µM CoroNa Green dye. The incubation time (2h) was constant in all experiments. M-R, The quantified Na^+ signal distribution in cell compartments in root zones of wheat varieties Purple Grain and Gladius. Mean ± SE (n = 21 − 43 cells). Please note that to avoid the image overexposure due to the use of 4 times higher concentration of CoroNa Green dye (80 µM), the Na^+ intensity of 20 µM dye incubated samples is lower than the one showed in Fig. 2, 3 and 4.

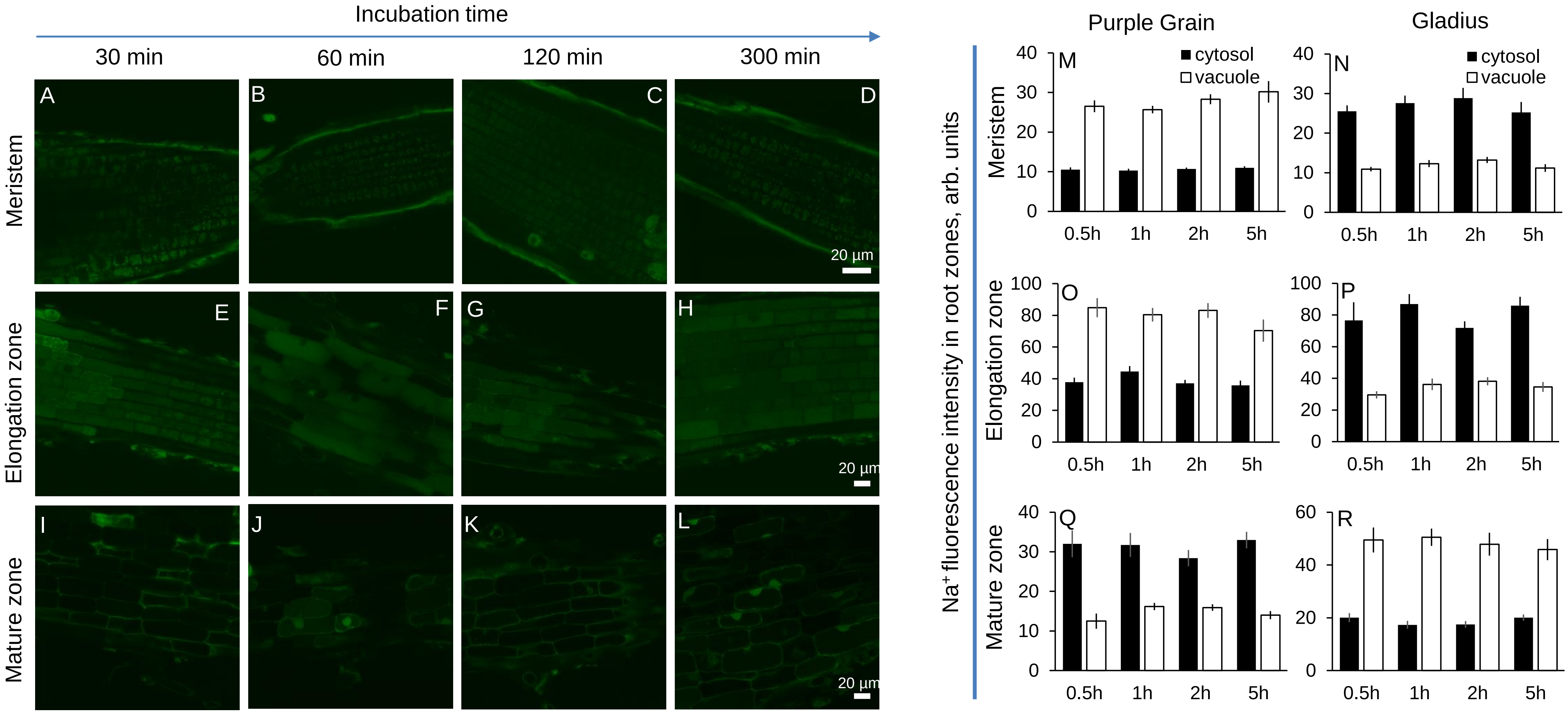

Figure S5. The effect of incubation time of CoroNa Green (20 µM) on the intensity of fluorescent Na^+ signal in wheat roots measured from different root zones. Hydroponically grown 4 days old wheat seedlings were treated with 100 mM NaCl for 72h. A-L, Representative (one of 4) confocal images of wheat variety (cv. Purple Grain) are shown for each zone for roots incubated with CoroNa Green dye (20 µM) with 30, 60, 120, 300 min. M-R, The quantified Na^+ signal distribution in cell compartments in root zones of wheat varieties Purple Grain and Gladius. Mean ± SE (n = 21 − 43 cells).

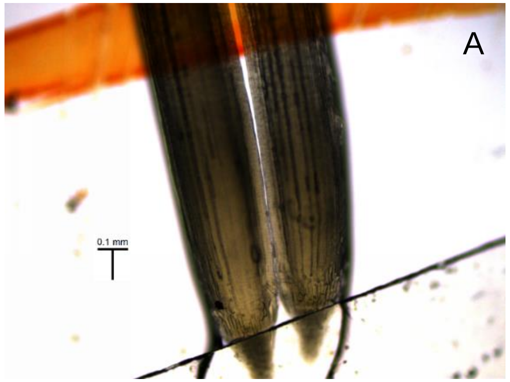

Before cutting

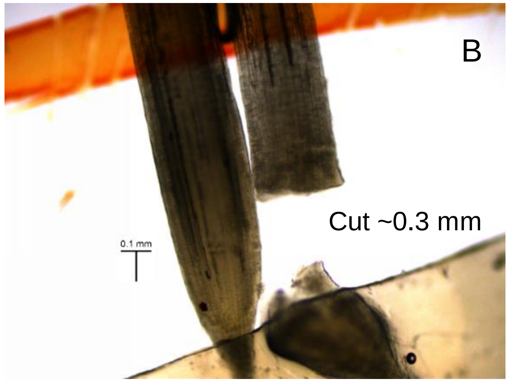

After cutting

Figure S6. Illustrating the procedure for removal of the root meristem tissue. The root meristem was carefully removed using a sharp scalpel blade under dissecting microscope from each of the seminal roots of wheat seedlings. A, Before the removal of the root meristem. B, After the removal of the meristem tissue from one seminal root. Hydroponically grown 4 days old wheat seedlings (cv. Kharchia 65) were used.

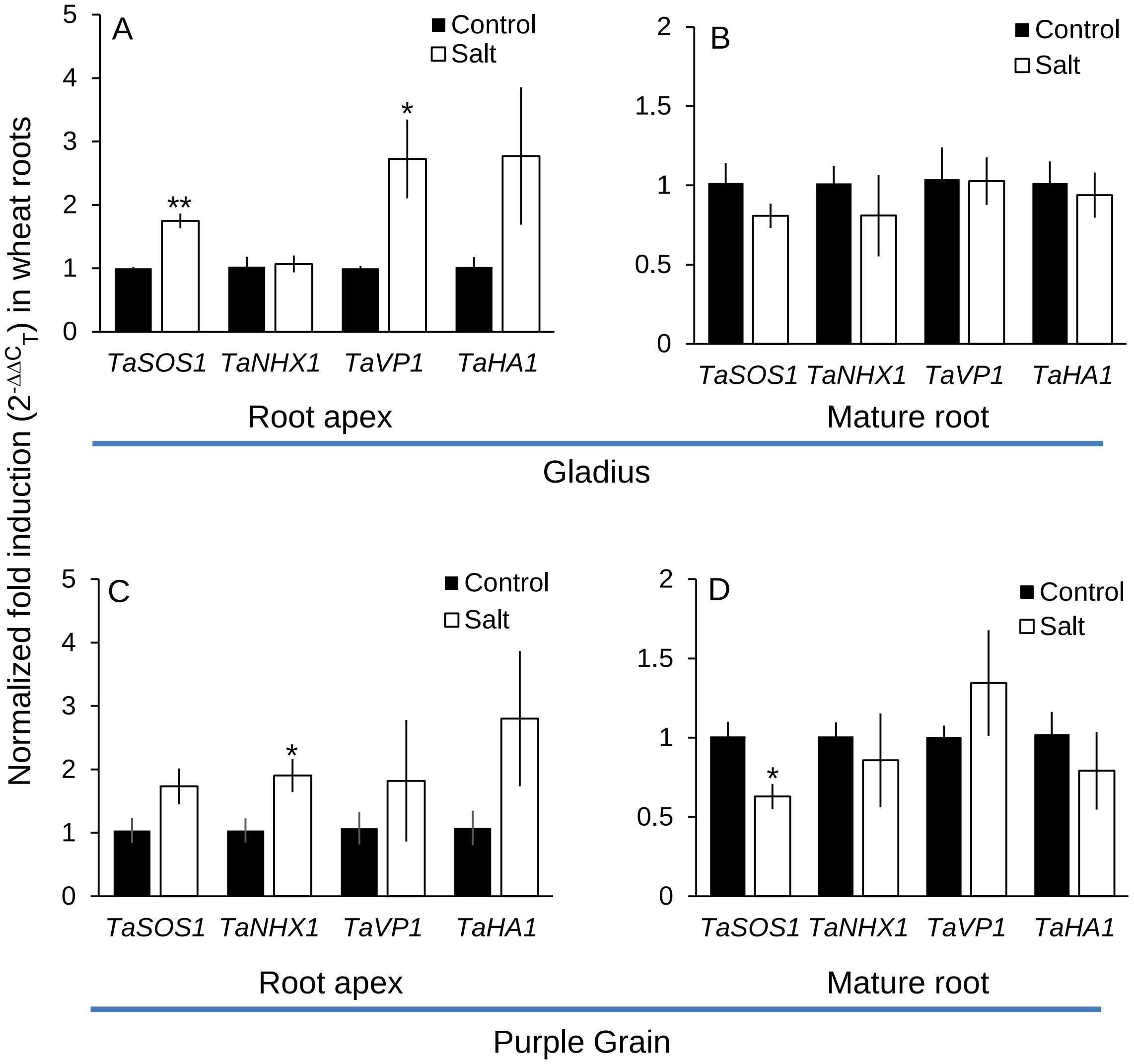

Figure S7. The relative gene expression patterns in root apex and mature root in bread (cv. Gladius) and durum wheat (cv. Purple Grain) after salt stress (100 mM NaCl, 24 h). A and B, Comparing transcriptional profiles of genes mediating Na^+ removal from the cytosol *TaSOS1*, *TaNHX1*, *TaVP*, and *TaHA1* in root apex (A) and mature root (B) in bread wheat (cv. Gladius). C and D, Comparing transcriptional profiles of genes mediating Na^+ removal from the cytosol *TaSOS1*, *TaNHX1*, *TaVP*, and *TaHA1* in root apex (C) and mature root (D) in durum wheat (cv. Purple Grain). Mean ± SE (n = 3 biological replicates). ** and * mean significant at $P < 0.01$ and $P < 0.05$, respectively.

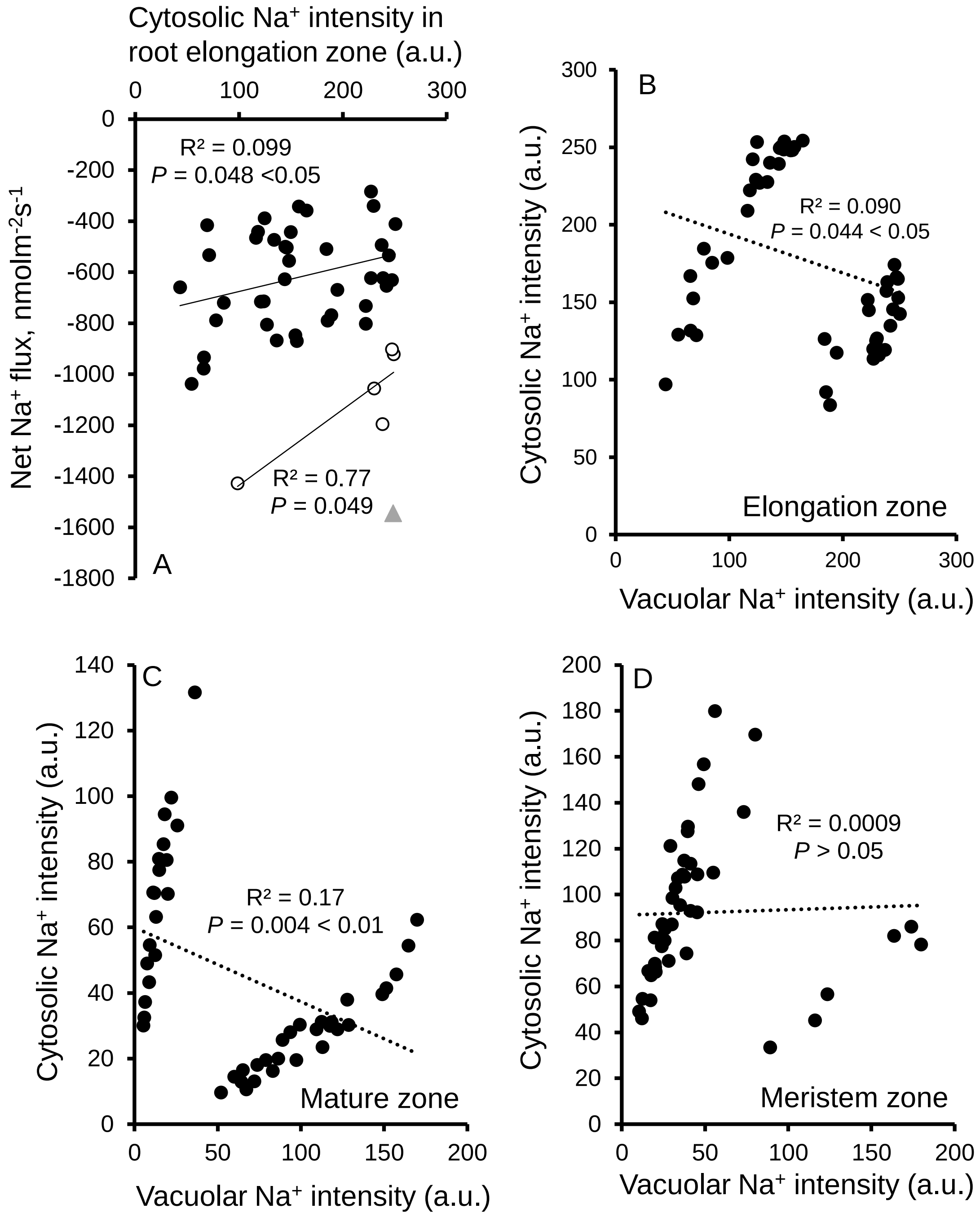

Figure S8. Correlations between cytosolic and vacuolar Na^+ intensity and Na^+ efflux. A, Correlation between cytosolic Na^+ intensity and Na^+ efflux in root elongation zone. B, C, and D, Correlations between cytosolic and vacuolar Na^+ intensity in root elongation, mature, and meristem zone, respectively.

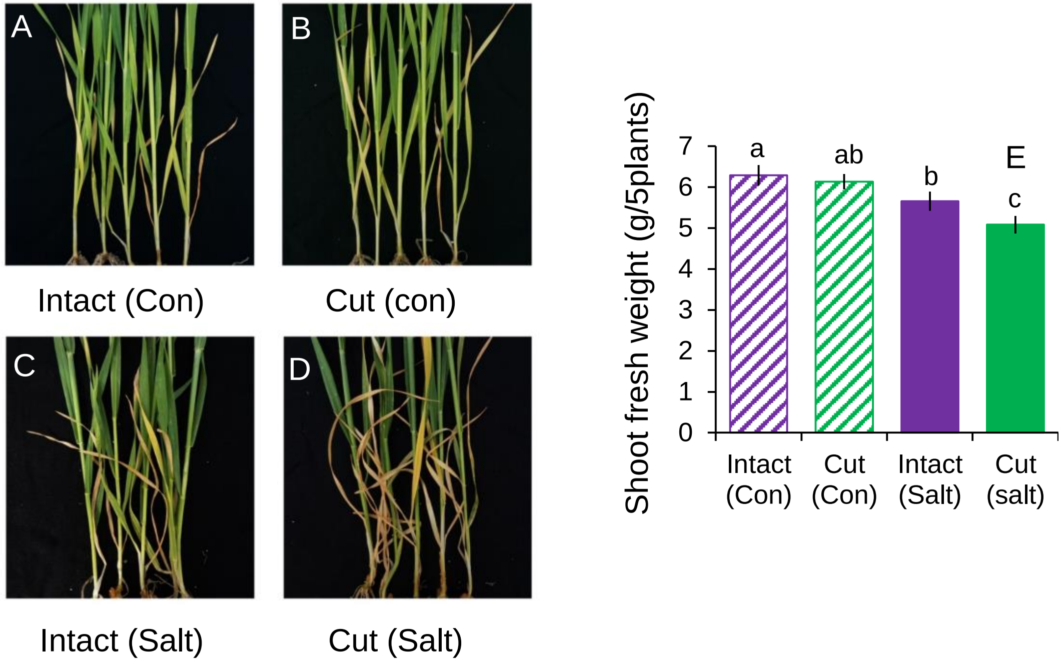

Figure S9. Removal of roots meristem results in a salt-sensitive phenotype. Bread wheat (cv. Westonia) seedlings with intact roots (intact plants) and those in which roots meristem were surgically removed at the age of 3 days old were used. Plants were grown hydroponically for the first 3 days. Immediately after removal of the meristem, seedlings were transferred into pots filled with the coarse sand/perlite (2:1 v/v). After one day adaption, plants (intact and tampered) were irrigated with 1/4 strength Hoagland solution with 100 mM NaCl (Salt) or without NaCl (Con) for 30 days. Panels A - D illustrate typical appearance of plants in each treatment. E, Shoot fresh weight of intact and cut plants. Mean ± SE (n = 8 to 10 plants). Different lower case letters denote significant difference between data at $P < 0.05$.

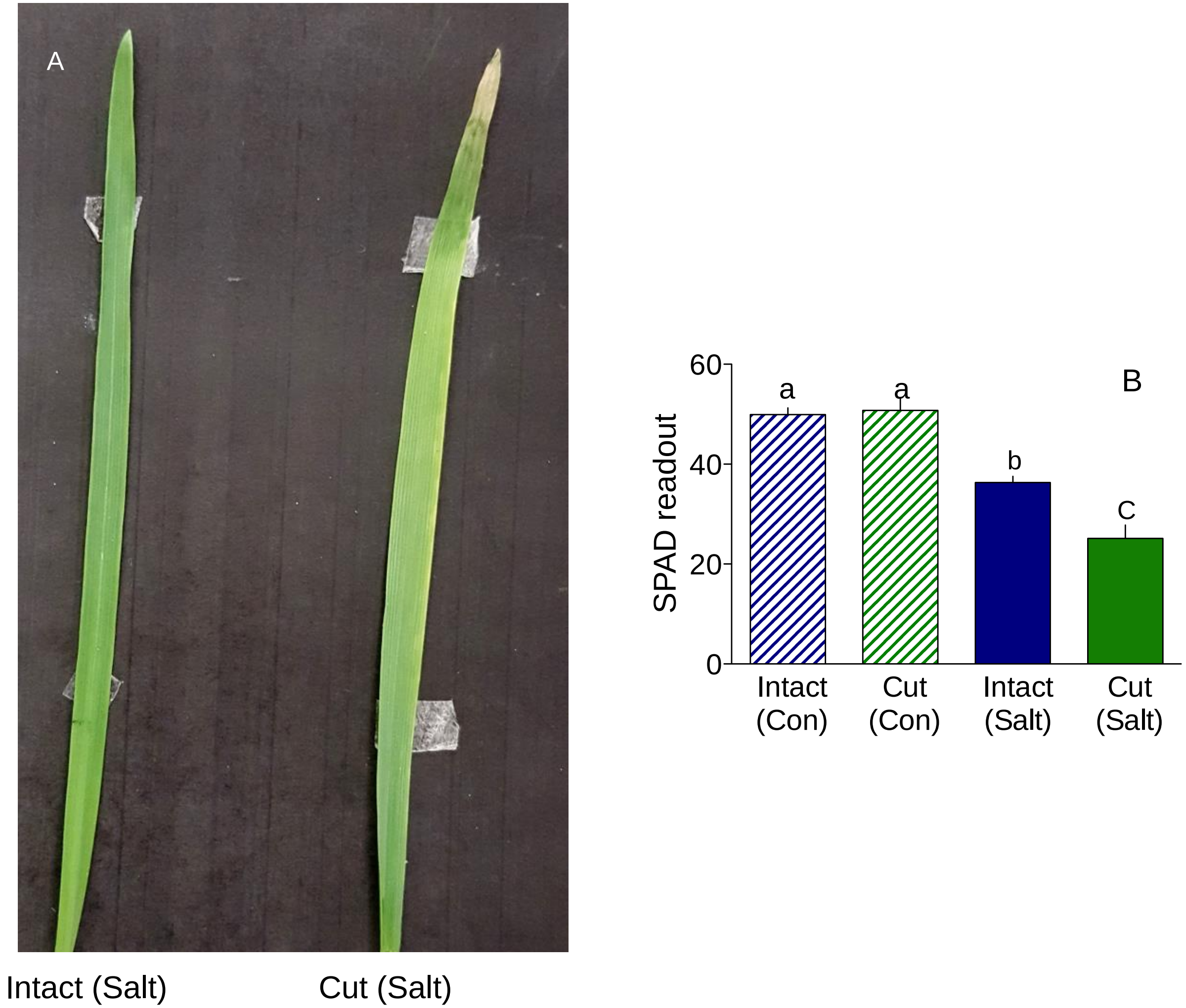

Figure S10. Removal of roots meristem results in higher vulnerability of durum wheat (cv. Wollaroi) to salt stress (200 mM NaCl, 10 days). A, Leaf phenotypic comparison between intact (with intact roots) and cut (in which roots meristem were removed) durum wheat plants after salt stress. B, Chlorophyll content between intact and cut durum wheat plants before and after salt stress. Mean ± SE (n = 5 plants). Different lower case letters denote significant difference between data at $P < 0.05$.

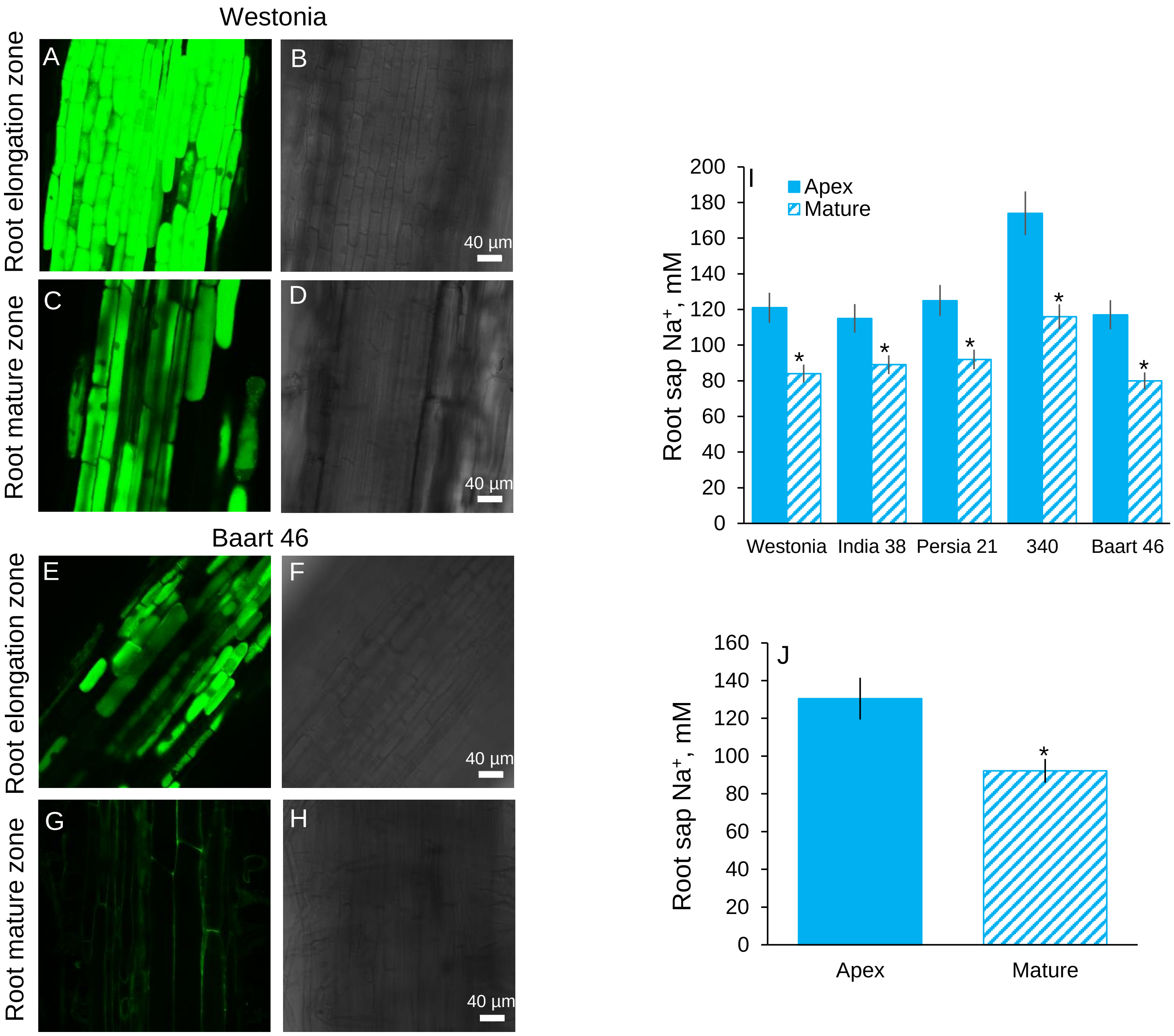

Figure S11. Sap Na^+ content in the root apical and mature tissues. Hydroponically grown 4 days old wheat seedlings were treated with 100 mM NaCl for 72h. A and C, Representative images of the root elongation zone (first 10 mm) and mature zone cells (30 - 40 mm) stained with CoroNa Green dye in salt-tolerant bread wheat cv. Westonia. Panels B and D show the corresponding light images. E and G, Representative images of the root elongation zone and mature zone cells stained with CoroNa Green dye in salt-sensitive bread wheat cv. Baart 46. Panels F and H show the corresponding light images. I, Sap Na^+ content in apical and mature root tissues of five wheat varieties. J, Averaged pooled values for sap Na^+ between root apex and root mature zone. Mean ± SE (n = 5). * and ** mean significant at $P < 0.05$ and $P < 0.01$, respectively.

Target genes	Locus	Primer sequences
TaNHX1	AY040245	F: 5’-GCCTGGTTCACCCATAGAGA-3’
		R: 5’-CACCGAAAGAATCCCAAGAG-3’
TaVP1	EU255237.1	F: 5’-CCTATCTTCGCCATTGCCTTC-3’
		R: 5’-CAGCATCCAGAGCATCAGTTC-3’
TaSOS1	AY326952	F: 5’-ATTCCCTCAGGTGCTTCGTG-3’
		R: 5’-TTTCCTCGAGCAACCCAGTC -3’
TaHA1	AY543630.1	F: 5’-GCTGATTGAGAAGGCTGATGG-3’
		R: 5’-TCGGTAAGCACAATGTCTGAAG-3’
TaActin	AF326781	F: 5’- TACACGAAGCGACATACAA-3’
		R: 5’-AATAGAGCCACCGATCCA-3’

Table S1. Primers used in this study.
